# A Self-Consistent Approach to Rotamer and Protonation State Assignments (RAPA): Moving Beyond Single Protein Configurations

**DOI:** 10.1021/acs.jcim.5c00859

**Published:** 2025-06-11

**Authors:** Mossa Ghattas, Prerna Gera, Steven Ramsey, Anthony Cruz-Balberdy, Nathan Abraham, Vjay Molino, Daniel McKay, Tom Kurtzman

**Affiliations:** † Ph.D. Program in Chemistry, The Graduate Center, City University of New York, New York, New York 10016, United States; ‡ Ph.D. Program in Biochemistry, The Graduate Center, City University of New York, New York, New York 10016, United States; § Department of Chemistry, Lehman College, City University of New York, New York, New York 10468, United States; ∥ Ventus Therapeutics, Inc., 4800 Rue Levy, Montreal, Quebec H4R 2P7, Canada; ⊥ Ventus Therapeutics U.S. Inc., 100 Beaver St. Suite 201, Waltham, Massachusetts 02453, United States

## Abstract

There are currently over 160,000 protein crystal structures obtained by X-ray diffraction with resolutions of 1.5 Å or greater in the Protein Data Bank. At these resolutions hydrogen atoms do not resolve and heavy atoms such as oxygen, carbon, and nitrogen are indistinguishable. This leads to ambiguity in the rotamer and protonation states of multiple amino acids, notably asparagine, glutamine, histidine, serine, tyrosine, and threonine. When the rotamer and protonation states of these residues change, so too does the electrochemical surface of a binding site. A variety of computational approaches have been developed to assign states for these residues by investigating all possibilities and typically deciding on a single rotamer or protonation state for each residue that is consistent with the crystal structure. Here, we posit that there are multiple rotamer and protonation states that are consistent with the resolved structure of the proteins and introduce a Rotamer and Protonation Assignment (RAPA) protocol which analyzes local hydrogen-bonding environments in the resolved structures of proteins and identifies a set of unique rotamer and protonation states that are energetically consistent with the experimentally reported crystal structure. We evaluate the RAPA-predicted configurations in molecular dynamics simulations and find that there are multiple configurations for each protein that maintain structures consistent with the X-ray results. In our initial evaluations of the RAPA protocol, we find that for most proteins (69/77) there are multiple energetically accessible rotamer and protonation state configurations however the total number is limited to 8 or fewer for most of the proteins (62 of 77). This suggests that there is no combinatorial explosion in the number of energetically accessible rotamer and protonation states for most proteins and investigating all such states is computationally feasible.

## Graphical abstract

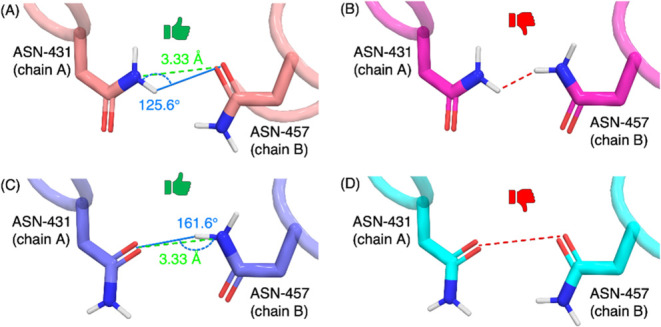

## 1 Introduction

Computational modeling of proteins in atomistic detail relies upon a precise set of coordinates of every protein atom. These are primarily obtained by X-ray crystallographic experiments, nuclear magnetic resonance (NMR), or cryo-electron microscopy (Cryo-EM). Currently, there are over 160,000 protein crystal structures obtained by X-ray diffraction with resolutions of 1.5 Å or greater in the Protein Data Bank (PDB),^1^ which accounts for 89% of the available protein crystal structures. At these resolutions, oxygen, nitrogen, and carbon atoms cannot be reliably distinguished from each other and hydrogen atoms do not resolve. This leads to ambiguities in the assignment of protonation and rotamer states which is particularly relevant for asparagine (ASN), glutamine (GLN), and histidine (HIS) residues. For asparagine and glutamine residues, there are two possible rotamer states for the side chain amide group. For histidine side chains, the imidazole ring has two possible rotamer states and three potential protonation states (being protonated in the delta, epsilon, or both positions) leading to six possible states that are not readily distinguishable from experimental X-ray scattering data. Though less frequent there can also be ambiguity in the protonation states aspartate (ASP) and glutamate (GLU) residues when they are uncharged. Similar ambiguity can be found in the positions of hydrogen atoms in hydroxy-containing residues serine (SER), threonine (THR), and tyrosine (TYR) as well as potential disulfide bridges between cysteine (CYS) residues.^2–4^

These are well-known limitations of structural determination experiments and a number of academic and commercial protein preparation methods have been developed to assign appropriate rotamer and protonation states to ambiguous scattering data. Rotamer assignment methods^5–9^ and corresponding tools^10–13^ are generally designed to identify configurations that minimize steric conflicts and optimize hydrogen-bonding networks. Protonation state tools^14–17^ rely upon p*K*_a_ predictors^18–24^ and hydrogen atoms are positioned to optimize hydrogen-bonding networks.^6,25,26^ A drawback of most methods of determining biomolecular structure from crystallographic data^27–29^ or preparing biomolecules for computational modeling,^30–37^ is that they focus on identifying a single *most-probable* configuration[Fn fn1] or require user input to modify the method’s proposed state assignments of the residues. Here, we posit that multiple configurations of a protein structure are consistent with the experimental data and introduce a Rotamer and Protonation state Assignment protocol (RAPA) that enumerates a set of unique configurations that are energetically consistent with the experimentally resolved structure. In the context of structure-based drug discovery (SBDD), enumerating such a set of viable rotamer and protonation (RP) states is particularly important. A fundamental principle of SBDD is that a potential drug must be electrostatically complementary to the protein surface i.e., donating or accepting hydrogen bonds (h-bonds) and making hydrophobic contacts where appropriate. Alternate rotamer states of residues in a binding site change the positions of h-bond contacts and alternate protonation states change the character of interaction sites from donor to acceptor or vice versa. Correspondingly, the chemical matter making complementary interactions changes with varying RP states. Most preparation approaches assign a single state. In virtual screening applications this effectively limits the screen to identifying chemical compounds that are complementary to that specific state. Identifying a set of viable RP states expands the chemical space being explored to chemical compounds that are complementary to any viable configuration. This, in turn, increases the ability of virtual screening workflows to identify compounds with potentially better affinity, pharmaco-kinetic properties, binding specificities, and patentability.

RAPA uses a recursive approach to determine the protonation and/or rotamer states of all ambiguous[Fn fn2] residues in the protein of interest. The approach works by estimating the energetics of the local hydrogen-bonding environment of all ambiguous residues in which all potential protonation and rotamer states of ambiguous neighboring residues are considered. The RAPA protocol is described in detail in Section 2.2. Unlike other approaches, RAPA often outputs multiple energetically viable configurations of a protein structure that are consistent with the experimentally determined X-ray diffraction data. We apply RAPA to a subset of 77 crystal protein structures from the Database of Useful Decoys–Enhanced (DUDE).^38^ In our initial evaluation of the protocol, RAPA identified 8 or fewer energetically accessible configurations for most of these proteins (62 of 77), nullifying the assumption that there could potentially be a combinatorial explosion of accessible configurations. This suggests that investigating all accessible RP states for most proteins is computationally feasible and that the selection of a single state, as most well-used protein preparation methods do, is arbitrary. We evaluate the proposed configurations using molecular dynamics (MD) simulations in two ways: First, we evaluate the stability of each configuration using simulations in which several core residues are restrained and determine whether the time-averaged MD structure remains comparable to the crystal structure. We find that in MD simulations of the RAPA proposed configurations, the proteins maintained structures that did not deviate considerably from the crystal structure with the vast majority of systems having RMSD’s deviating less than 2.0 Å from the experimental structures when calculating the RMSD for 90% of the atoms with the lowest B-factors. Second, for ASN, GLN, and HIS residues that are predicted to be stable in multiple rotamer states, we evaluate whether both predicted states are stable. We find that for most systems (70 of 77) there are multiple configurations that are consistent with the X-ray scattering data, evaluated to be energetically feasible, and have stable structures in MD simulations.

## 2 Methods

### 2.1 Selection of Proteins for Analysis and Initial Preparation

We applied RAPA to the subset of 77 protein structures (Table S1) from the DUDE data set that have no missing loops, covalently bound small molecules, or heme groups. Each protein was prepared using OpenEye Spruce^37^ using default parameters. Ligand and water molecules were removed from all systems prior to being analyzed.

### 2.2 RAPA Program

#### 2.2.1 RAPA Workflow

The RAPA workflow is diagrammed in Figure 1.

**Figure 1:**
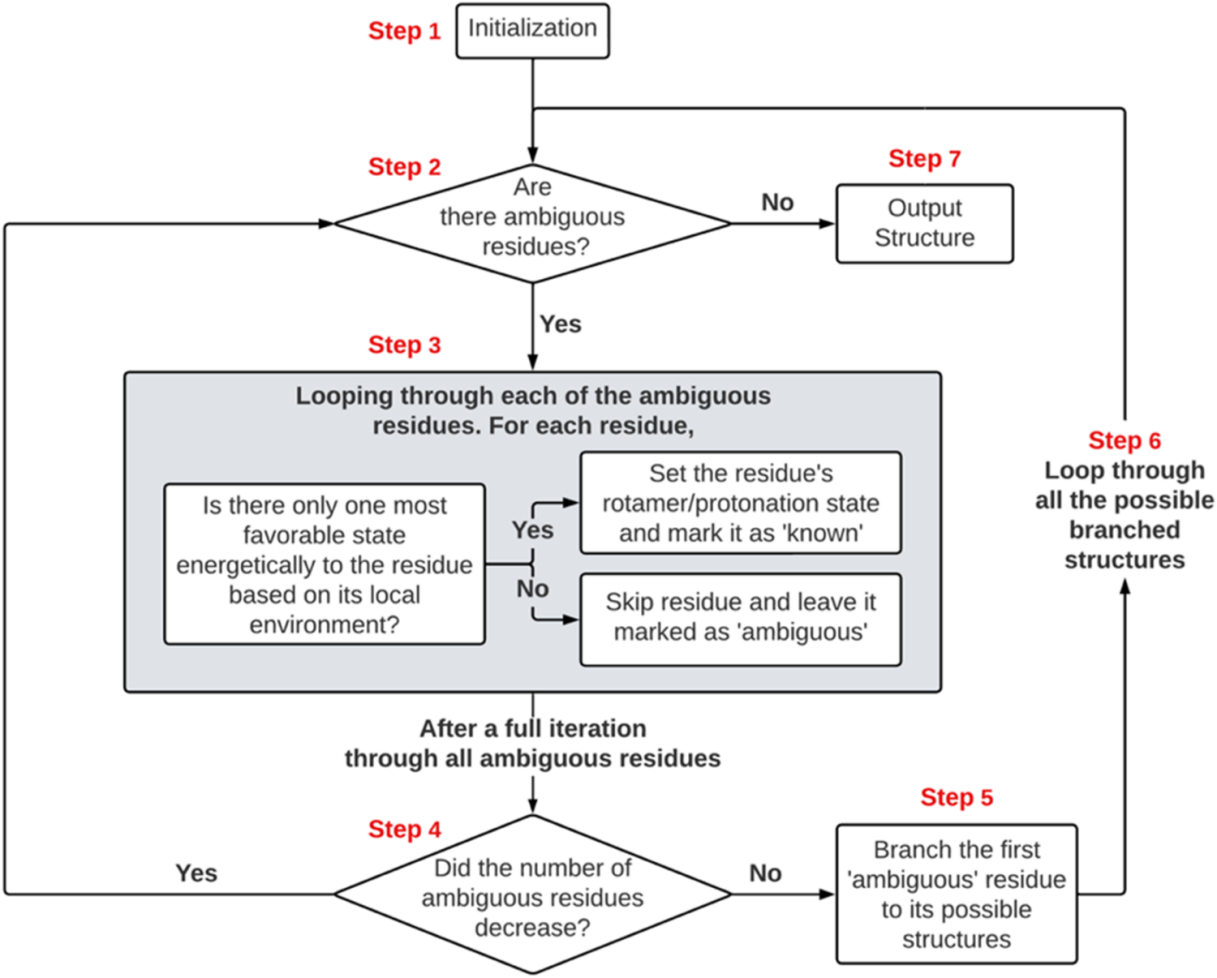
Flowchart demonstrating the sequence of operations performed by the RAPA methodology. The process begins with an input protein structure, and it terminates with one or more RAPA states output in PDB format.

##### 2.2.1.1 Step 1 (Initialization)

Step 1 assigns *known* status to all polar heavy atoms for which there is no ambiguity. Backbone nitrogen atoms, arginine, and tryptophan side chain nitrogen atoms are always h-bond donors. Backbone carbonyl oxygen atoms are always h-bond acceptors. Cysteine sulfur atoms are either protonated or deprotonated if determined to be involved in disulfide bond. All of these are assigned *known* status at initialization. All other side chain heavy atoms that can act as donors or acceptors (N and O) are assigned *unknown* status. Residues with side chains that contain an *unknown* heavy atom or for which there are possible alternate rotamer states are considered *ambiguous*.

##### 2.2.1.2 Step 2 (Termination Check)

If there are *ambiguous* residues present in the structure then the procedure continues to step 3. If there are no *ambiguous* residues, the structure considered fully determined and proceeds to Step 7.

##### 2.2.1.3 Step 3 (Environment Evaluation)

Each *ambiguous* residue is sequentially investigated. For each residue, the energetics of all its possible RP states as well as all RP states of *proxima*l neighbor residues are estimated (Section 2.2.5). If one RP state is found to be energetically more favorable than all other possible states then that RP state for the residue is set and the status of the residue is switched from *ambiguous* to *known*. Otherwise, the status of the residue is maintained as *ambiguous*. In either case, the next residue is then investigated. Step 3 is concluded after all *ambiguous* residues have been investigated. Details of the energetic estimates are fully described in Section 2.2.3. If a residue has no *proximal* neighbors then it is considered *fully solvated* and set as *known* and maintains the assignments as input.

##### 2.2.1.4 Step 4

If the number of *ambiguous* residues changed in Step 3, the protocol returns to Step 2 and proceeds from there. Otherwise, the procedure advances to Step 5.

##### 2.2.1.5 Step 5 (Branching)

If this step is reached, then the protocol has determined that there is at least one residue with two or more RP states that are deemed to be consistent with the crystal structure. We term these as *“degenerate”* states which consist of the RP state with the lowest assessed energetic environment (Section 2.2.5) and all other RP states that have an environmental energy within a user defined cutoff of this lowest value. For the evaluation purposes reported here, we set this cutoff to be 1 kcal. At this step, the first *degenerate* residue in sequence is identified. A copy of the system (*known* and *ambiguous* status) is made for each *degenerate* RP state of the residue in which that RP state is assigned as *known*. The protocol then proceeds to step 6. We refer to this process as *branching* and each copy as a *branch*.

##### 2.2.1.6 Step 6

A loop is initiated in which each branched configuration is processed through the protocol with each branch being independently processed initiating at step 2 to check if there are remaining *ambiguous* residues. Each new configuration continues the process until all residues are assigned *known* RP states with no remaining *ambiguous* residues in which case the configuration is entirely determined and output in PDB format (Step 7).

##### 2.2.1.7 Step 7

The configuration is entirely determined and output into PDB format and the workflow for this *branch* is terminated.

#### 2.2.2 Treatment of the Different Residue Types

##### 2.2.2.1 Known Residues

The following residues are assigned as *known* in the initialization (STEP 1). Nonpolar residues such as Glycine (GLY), Alanine (ALA), Valine (VAL), Leucine (LEU), Isoleucine (ILE), Methionine (MET), Proline (PRO), and Phenylalanine (PHE) as they have no side chain donors or acceptors. Tryptophan (TRP) side chain nitrogen atoms are always considered protonated. Lysine (LYS) and Arginine (ARG) side chain nitrogen atoms are always assigned the positively charged protonation state. Cysteine sulfur atoms are protonated unless they are within 2.3 Å of a neighboring CYS sulfur atom in which case they are deprotonated set to be participating in a disulfide bond. In either case the CYS residue is assigned *known* status.

##### 2.2.2.2 Histidine Residues

HIS residues are initially assigned *ambiguous* as there is uncertainty in both their protonation and rotamer states. Local energetics are evaluated for the six RP states which are all combinations of the 2 possible rotamer states and 3 protonation states (HIE, HID, and HIP).

##### 2.2.2.3 Asparagine and Glutamine Residues

ASN and GLN residues are initially assigned as *ambiguous* as there is uncertainty in their rotamer states. Local energetics are evaluated for both possible rotamer states for these residues.

##### 2.2.2.4 Aspartate and Glutamate Residues

ASP and GLU residues are initially assigned *ambiguous* in their protonation state. They are considered negatively charged (unprotonated) unless they are part of an acid dyad. If two ASP or GLU residues are proximal to each other, defined as having carboxylate oxygen atoms within 3.8 Å, then the energetics of all possible singly protonated configurations is evaluated (Figure S2 in Supporting Information). If one is concerned that there may be internal ASP/GLUs that are protonated,^39^ the protocol can be modified such that ASP/GLU residues are initially set as unknown however the protocol was evaluated without this consideration.

##### 2.2.2.5 Serine, Tyrosine, and Threonine Residues

SER, THR, and TYR residues are initially assigned to be *ambiguous.* Their hydroxy groups can act as both donors and acceptors, however, there is uncertainty in the position of the proton which determines the directionality of the donor and acceptor interactions. For SER and THR residues local energetics are evaluated for 9 potential positions of the hydroxy hydrogen. The hydrogen atom with the shortest distance from the neighboring heavy atom is the position of the hydrogen atom evaluated energetically. The 9 possible positions include the three staggered positions corresponding to their optimal sp3 hybridization as well (±20° rotations) from each of these three positions (see Figure 4). The hydroxy groups of TYR residues are planar about the aromatic ring due to bond resonance. The energetic evaluations position the hydroxy group’s H atom at 2 distinct positions that are trigonal-planar with the aromatic ring carbon consistent with SP2 hybridization.

#### 2.2.3 Local Environment Energy Estimations

Energetics of h-bonds and electrostatic clashes are evaluated only for residues for which the distance between polar heavy atoms is *proximal* which we define here to be within 3.8 Å.

Residue–residue hydrogen-bond energies are estimated using a lookup table derived from water–water hydrogen-bond energies parameterized based on heavy atom–heavy atom (hv–hv) distances and heavy atom–hydrogen–heavy atom (hv–h–hv) angles. The lookup table was constructed using a potential energy surface (PES) calculation generated using Schrodinger Jaguar^40^ on a set of configurations of a water dimer with Oxygen–Oxygen (O–O) distances ranging from 2.5–4.0 Å with 0.1 Å intervals and O···H–O angle ranging from 90–180° with 0.1° intervals. Local h-bond energies for the protein use the hv-hv distance and hv–h–hv angle as a proxy for the O–O distance and O···H–O angle. Full details of the Jaguar calculations for constructing the lookup table and a plot of generated potential energy surface (Figure S1) can be found in the Supporting Information.

The energy of electrostatic clashes (e.g., between two h-bond donors or two h-bond acceptors) is simply estimated to be the negative of the energy of making a favorable h-bond interaction between donor–acceptor pairs. This is illustrated in Figure 2 for which the estimated energetics of the O–O clash (Figure 2, left) is simply estimated to be the inverse of the favorable Amino-Carbonyl interaction in the same position (Figure 2, right).

**Figure 2:**
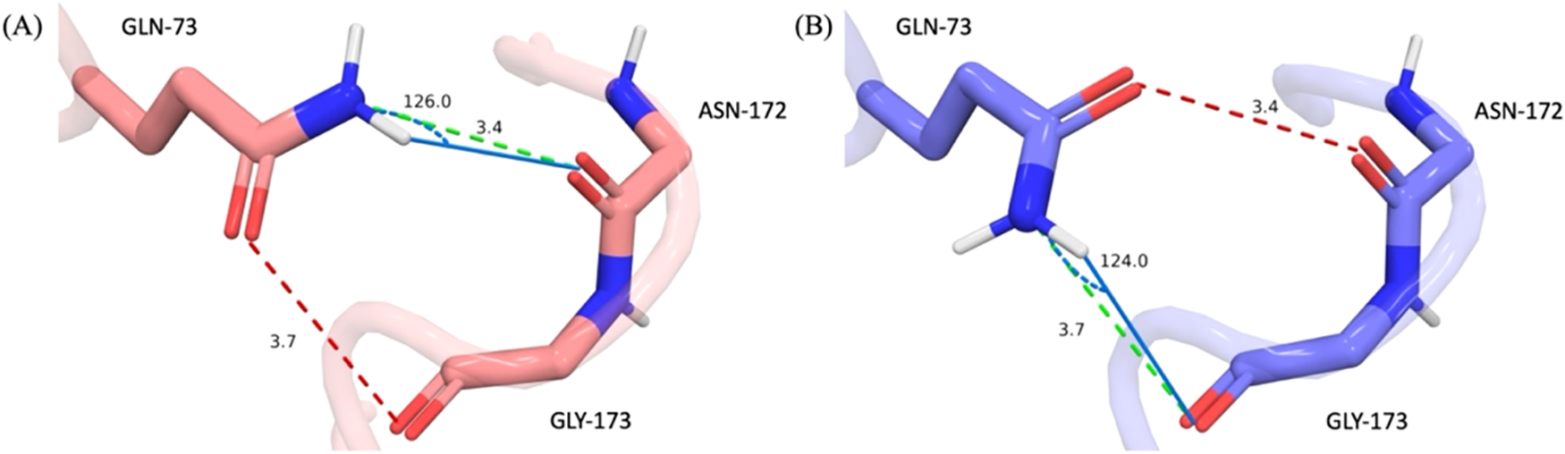
Interactions of β-site amyloid cleaving enzyme GLN-73 (PDB entry 3L5D^41^) Here we evaluate the local energetics of two possible rotamer states of GLN-73. In each state, GLN-73 forms a h-bond interaction (favorable, green dashed lines) and an electrostatic clash (unfavorable, red dashed lines). The left configuration was found to have lower total energy as the h-bond is stronger than the electrostatic clash. However, the difference in local energies for the two rotamer states of GLN-73 (A, B) is estimated to be 0.76 kcal/mol. Thus, in our evaluation using an energetic cutoff of 1 kcal/mol, GLN-73 is considered degenerate and both RP states are further evaluated in the protocol.

The energetics of a given RP state is estimated by summing up all local polar interactions as shown illustrated in Figure 2.

#### 2.2.4 Evaluation of Residue RP States

In step 3 of the workflow, each *ambiguous* residue is sequentially investigated such that the local energetics are tabulated for each potential RP state in each environment provided by all possible configurations of proximal residues.

As an example of this, consider an RP state from the human dopamine D3 receptor (D3R, PDB: 3PBL^35^) that contains two residues ASN-1132 and SER-1117 (Figure 3) with the amide of the ASN interacting with the hydroxy of the SER. ASN-1132 has two possible rotamer states and the SER-1117 hydroxy group can interact with ASN-1132 as either a h-bond donor or acceptor resulting in four possible RP states.

**Figure 3:**
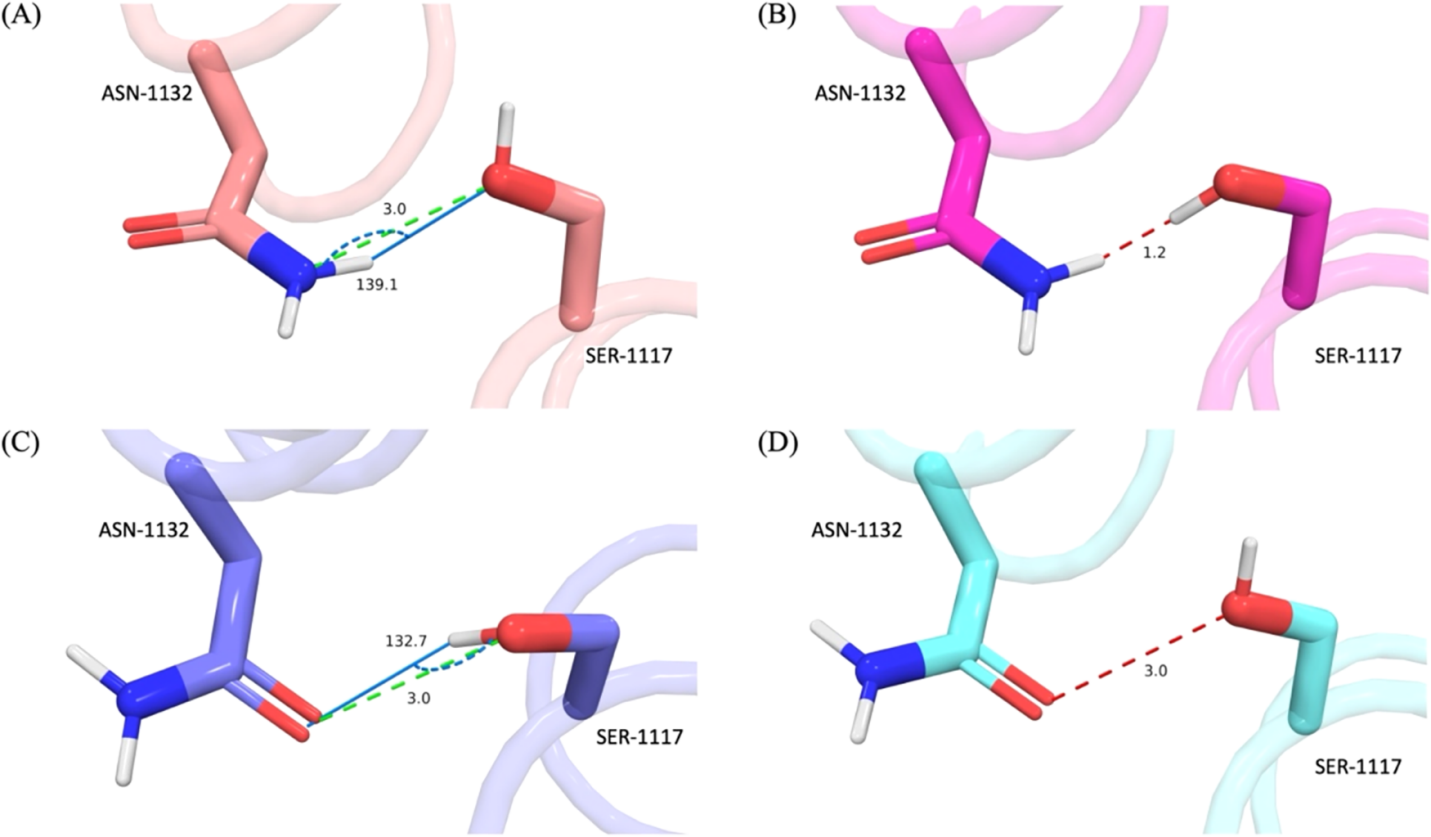
Interactions of Dopamine receptor 3 residues ASN-1132 and SER-1117 (PDB entry 3PBL^42^). (A–D) show the 4 environments evaluated by RAPA. Those 4 environments arise from 2 rotamer states of ASN-1132 and 2 possible states of SER-1117. Electrostatic clashes shown as red dashed lines, h-bond interactions shown as green dashed lines. H-bond angles displayed as marine line and arc. The difference in local energetics between environments (A, C) was found to be 0.86 kcal/mol, thus, both environments are deemed energetically viable (1 kcal/mol cutoff).

Of these 4 possible configurations, two are unfavorable (Figure 3b,d) containing electrostatic clashes and two are favorable forming h-bonds between the two residues (Figure 3a,c). The two favorable configurations have energetics estimated to be within 0.85 kcal/mol and thus, using a cutoff of 1 kcal/mol, the two RP states of ASN-1132 are considered to be energetically *degenerate.*

When the hydroxy of SER-1117 donates a h-bond to the ASN-1132 amide oxygen, 9 possible positions of the hydroxy hydrogen are investigated in the energy assessment (Figure 4). The hydrogen position used for the energy evaluation is the position that results in the shortest h-bond distance to the oxygen acceptor. In this case, the hydrogen atom labeled P7 makes the shortest distance to the amide oxygen of ASN-1132 (Figure 3c).

**Figure 4:**
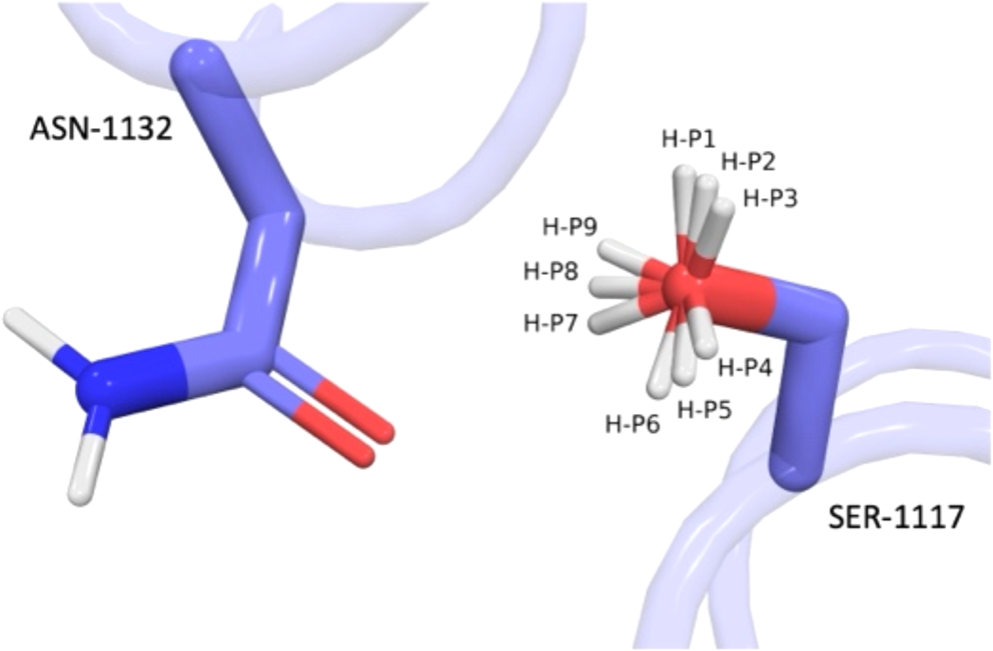
Determining the hydroxy directionality of SER-1117 when it donates a h-bond to ASN-1132 amide oxygen. The 9 potential positions of the hydrogen atom are labeled H–P1 to H–P9. In this example H–P7 is the shortest distance to the oxygen acceptor and is used in the energy evaluation.

#### 2.2.5 Example of Branching to Alternate Configurations

Here, we illustrate how the *branching* into separate configurations occurs in Step 5 when an RP state is *degenerate.* Figure 5 shows the interactions of two *ambiguous* asparagine residues from Estrogen receptor β (PDB entry 2FSZ^43^). In every evaluation of Step 3, both rotamer states are considered *degenerate* as the configurations in Panel A and C are within 1 kcal of each other. Eventually, at Step 5, two configurations of the protein are branched. In the first branch, ASN-431 of chain A is set to *known* in the configuration shown in panel A. In the second branch, the configuration is set to be *known* with the configuration shown in panel C. Each branch is fed through the protocol separately. For the first branch, when ASN-457 of chain B is investigated, its two rotamer states are evaluated interacting with ASN-431 of chain A and the possible states are Figure 5a,b. It is found that the energy of A is more than 1 kcal more favorable than B and for the first branch, ASN-431 (chain A) and ASN-457 (chain B) are *known* in the configuration shown in panel A. The same procedure for the second branch yields configuration of C as *known* for both residues. In this case, configurations representing both panels A and C are represented in the set of final configurations output by the protocol.

**Figure 5:**
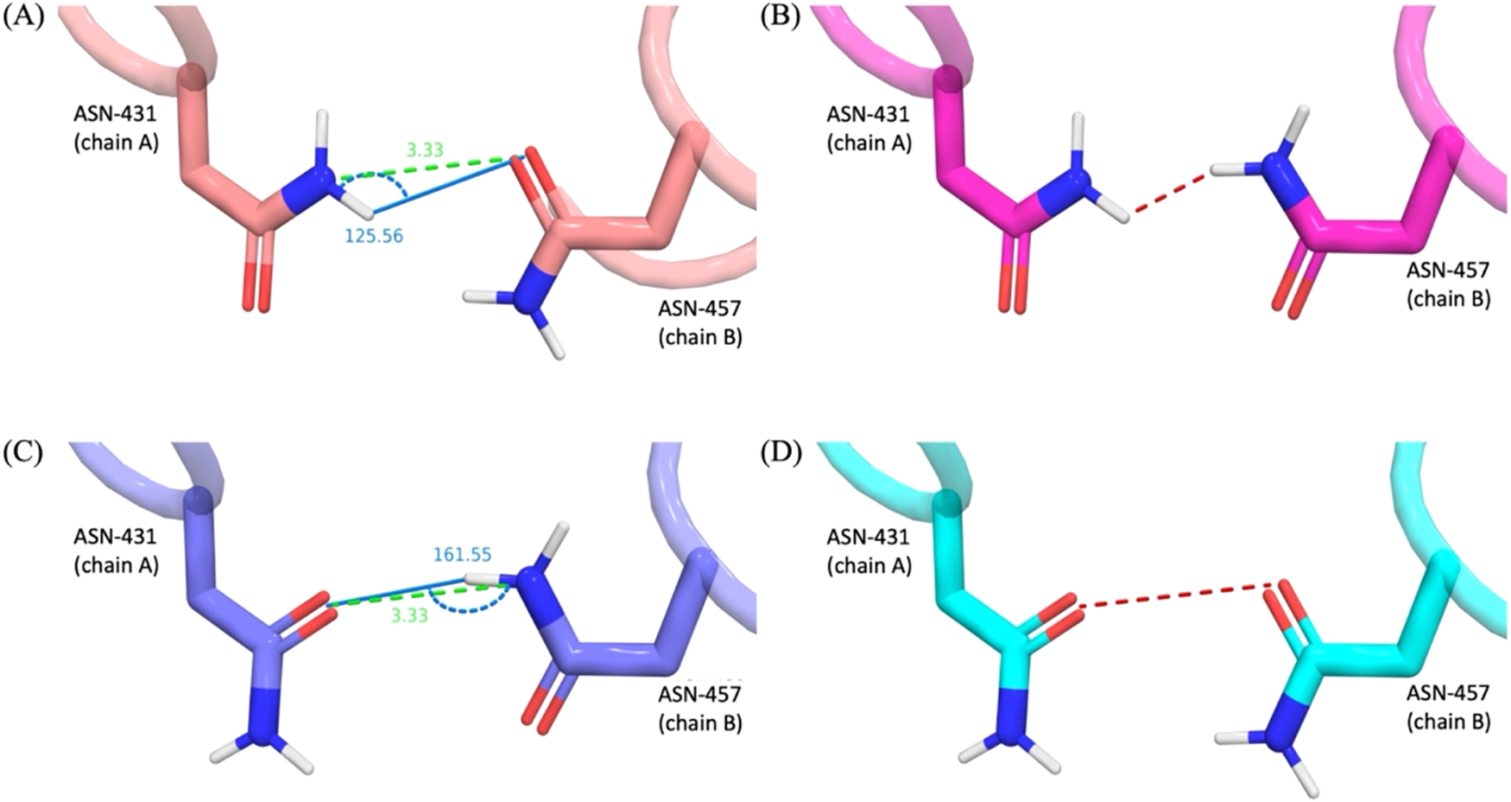
Potential interactions of two ambiguous asparagine residues from Estrogen receptor β (PDB entry 2FSZ^43^). Here, each asparagine has two possible rotamer states yielding four possible configurations shown in the four panels. Electrostatic clashes shown as red dashed lines, h-bond interactions shown as green dashed lines. H-bond angles displayed as marine line and arc. The energy of each proximal interaction is estimated using the angle and distance in a precalculated lookup table. In this case, the configuration in Panel C is the lowest energy and the configuration in Panel A is within 1 kcal of this value so the residue remains ambiguous.

### 2.3 Molecular Dynamics Simulations

We conducted two sets of MD simulations to evaluate RAPA proposed configurations. The first set (Section 2.3.2.1) evaluated whether a proposed configuration complexed with the cognate ligand remained stable in MD simulations. In these simulations, a subset of the core α carbons of the protein with low reported B-factors were restrained however the rest of the protein remained unrestrained. These simulations were designed to assess whether RAPA proposed configurations led to steric or electrostatic clashes that resulted in unstable MD simulations and to assess whether the unrestrained parts of the protein deviated substantially from the crystal structure. We restrain the core atoms to prevent global translation and rotation of the protein which avoids the need to align the protein when investigating regions of the protein surface. We do this because alignments of the protein distort solvent density distribution in a manner that depends on the alignment method. We therefore find that these restraints give a more accurate representation of the water structure on the surface. We also ran unrestrained simulations for the same validation and report the results in Supporting Information (Figure S6).

The second set of simulations (Section 2.3.3.1) evaluated *ambiguous* residues for which RAPA proposed two or more viable rotamer states. These simulations were designed to assess whether the proposed rotamer states were stable in MD simulations. In these simulations, the side chains being investigated were allowed to rotate freely allowing their rotamer states to interchange while the rest of the protein was restrained. This was an evaluation as to whether the proposed rotamer states were consistent with the experimental crystal structure.

#### 2.3.1 Initial Preparation and Force Field Parameterization

Small molecule ligands were parameterized using version 22.0 for both Amber^44^ and Ambertools.^45^ Atom types were assigned with *antechamber*,^46^ with AM1-BCC^47^ as the charge model, and “General Amber Force Field 2” (GAFF2 ^48^) as the ligand force field. For the two sets of MD validations, *tleap* ^45^ was used to prepare and solvate the systems with protein force field ff99SB^49^ and OPC^50^ water in a rectangular polyhedral box with a 10 Å buffer between the box edges and the protein.

#### 2.3.2 Complex Stability Simulations

##### 2.3.2.1 Molecular Dynamics Protocol

The MD preparation followed the protocol described in our previous work.^51^ The systems were energetically minimized in a two-step process. The first minimization step was performed with 1500 steps of steepest descent with all protein atoms restrained harmonically using a force constant of 100 kcal/mol·Å^2^. For the second minimization step, only the protein heavy atoms were restrained for 1500 steps. The system was heated from 0 to 300 K in a 240 ps NVT simulation with the protein heavy atoms restrained with the same force constant; the temperature was regulated by Langevin thermostat with collision frequency of 1 ps. This was followed by a 20 ns NPT equilibration simulation with the atom restraints uniformly reduced from 100 to 2 kcal/mol·Å^2^ over 10 ns. The output from this was used as the input configuration for the production phase MD simulations. In the production phase, the temperature was regulated via a Langevin thermostat set to 300 K with a collision frequency of 2 ps. The pressure (1 atm) was maintained by isotropic position scaling with a relaxation time of 0.5 ps.

During the 10 ns production phase, the protein–ligand complex was simulated with restraints applied to the α carbons of three disparate residues. These restrained α carbons had low B-factors and were located at least 10 Å away from the crystallized ligand. We manually selected the three restraint sites from different α helices. If there were not enough α helices, we chose β sheets, and if neither were available, we selected low B-factor residues from the protein core. The restraints applied had a Cartesian weight of 2 kcal/mol·Å^2^, which prevented the protein from rotating or translating. This protocol,^52^ which we will refer to as 3 distal site restraints (3DS), was designed to prevent global translation and rotation of the protein while allowing structural fluctuations.

##### 2.3.2.2 Postprocessing and RMSD Analysis

For each MD simulation, we used CPPTRAJ^53^ to generate the time-averaged structure of the 40,000 snapshots in the trajectory. The time-averaged structure refers to a representative structure in which the reported atom positions are the average position over all snapshots. The time-averaged MD structure was aligned to the crystal structure with CPPTRAJ and the RMSD was calculated from the distances between the backbone heavy atoms (Atom names in Amber: N, CA, and C) between these two aligned structures. In this analysis, atoms that have no B-factor data were excluded. RMSDs are reported for subsets of atoms with each subset consisting of the atoms with the lowest 50, 70, and 90% of B-factor values. We refer to these subsets as *RMSD-50%*, *RMSD-70%*, and *RMSD-90%* respectively. We perform the RMSD analysis on these subsets to exclude floppy regions of the protein with high B-factor values which can contribute disproportionately to RMSD calculations.

#### 2.3.3 Rotamer States Simulations

##### 2.3.3.1 Molecular Dynamics Protocol

The protocol of this MD set is the same as described in the previous section (Section 2.3.2.1) except for the following: the 20 ns NPT equilibration reduces the atom restraints from 100 to 0.10 kcal/mol·Å^2^ over the first 10 ns. In the 10 ns production runs, Cartesian restraints are applied on all protein heavy atoms except for the side chains of the *ambiguous* residues being evaluated, such as amide groups of ASN/GLN residues and the imidazole rings of HIS residues. The production run restraints strengths are set to 0.1 kcal/mol·Å^2^. This maintains the crystal three-dimensional (3D) structure but allows these residues’ amide groups and imidazole rings to rotate.

##### 2.3.3.2 Molecular Dynamics Analysis

We use CPPTRAJ^53^ to extract the dihedral angle distribution of the side chains of ASN, GLN and HIS across the trajectories. The dihedral angles in Amber atom names are CA-CB-CG-OD1 for ASN residues, CB-CG-CD-OE1 for GLN residues, and CA-CB-CG-ND1 for HIS residues. We fit each dihedral angle distribution to Gaussian functions and a Gaussian curve representing a rotamer state was considered significant if its population was more than 10% of the simulation. To examine the sampling and potential interconversion between possible rotamer states, the distributions of dihedrals were overlaid in a single plot for each residue.

#### 2.3.4 Labeling Ambiguous Residues

When the procedure is completed for a given protein, one of three labels is assigned to each ASN, GLN, and HIS residue. A residue is labeled as *fully solvated* if it makes no h-bond interactions with the protein. If a residue makes one or more h-bond interactions with the protein and no alternate RP states have energies within 1 kcal of the minimum energy state, then it is labeled as *fixed*. Finally, a residue is labeled as *degenerate* if it has multiple RP states that are within 1 kcal of the minimum energy RP state.

## 3 Results

### 3.1 RAPA Proposed Alternate Configurations

In the 77 proteins investigated here, there were a total of 2274 ASN/GLN residues and 780 HIS residues. We find that the majority of these are *fixed* (84% of ASN/GLN and 89% of HIS) with only one energetically viable RP state. However, we find that a small percentage of the ASN/GLN (4%) and HIS residues (10%) have *degenerate* RP states. The remainder of the residues are *fully solvated* and make no intramolecular h-bond interactions (Table 1).

**Table 1:** RAPA Labels of the ASN, GLN, and HIS Residues in the 77 Structures

total # of residues	# *degenerate* residues	# *fixed* residues	# *fully solvated* residues
2274 ASN/GLN	87	1918	269
780 HIS	79	694	7

We found that 14 systems (18%) had one configuration and 68 (88%) of the systems had 32 or fewer configurations. All of the 9 systems with more than 32 configurations were homodimers, -trimers, and -tetramers and for each monomer the number of proposed configurations was 24 or fewer (Figure 6).

**Figure 6:**
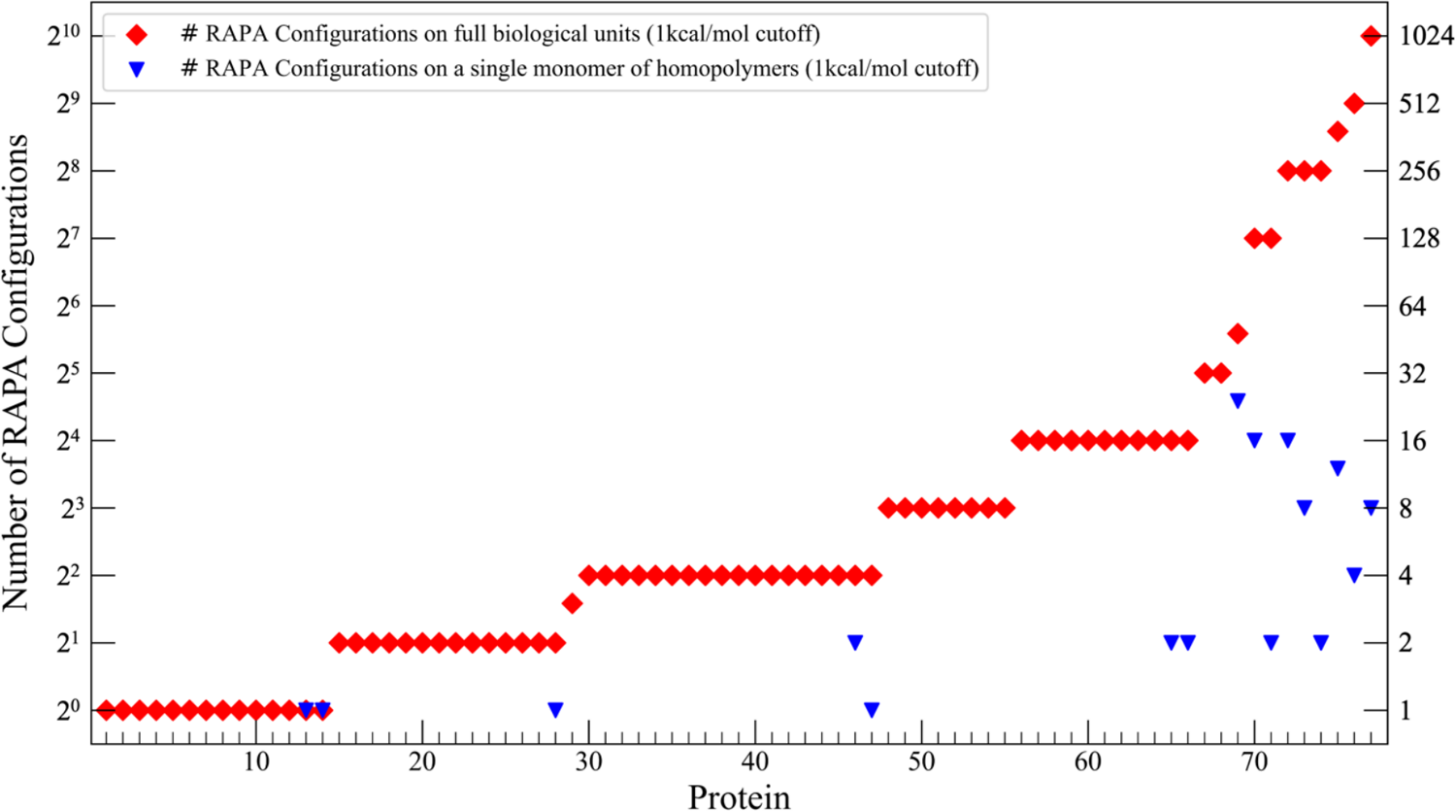
Total number of unique configurations determined by RAPA for 77 protein systems when the full biological unit of the system is evaluated (red diamonds) versus when one single repeating monomer is considered for systems containing homopolymers (blue triangles). Data is for a 1 kcal/mol energy cutoff. Data for an energetic cutoff of 2 kcal/mol can be found in Supporting Information, Figure S7.

### 3.2 Molecular Dynamics Simulations of the RAPA Proposed Structures

#### 3.2.1 RAPA State Configurational Stability

For each protein target that had 48 or fewer proposed configurations (Figure 5), we ran 10 ns MD simulation using the 3DS protocol (Section 2.3.2.1) for each configuration. The restraints of the 3DS protocol^52^ were constructed to prevent global rotation and translation of the protein while otherwise allowing protein structural fluctuations.

These simulations assessed whether the RAPA proposed configurations were stable in MD simulations (i.e., did not crash due to large forces) and whether the protein maintained structures that did not deviate substantially from the crystal structure. All 469 MD simulations completed their entire 10 ns simulations did not crash.

For each of the 469 simulations, we calculated the RMSD between the time-averaged structure and the crystal PDB structure as outlined in the methods sections. For all systems for the *RMSD-90%* subset of atoms, the RMSD was 2.18 Å or under and 467/469 systems had values of under 2.0 Å (Figure 7). Fifty-six percent of the simulations had RMSDs of 1.0 Å or lower including many systems with multiple configurations (Figure 8). RMSD data for the *RMSD-50%* and *RMSD-70%* subsets of atoms in the Supporting Information (Figures S3–S5).

**Figure 7:**
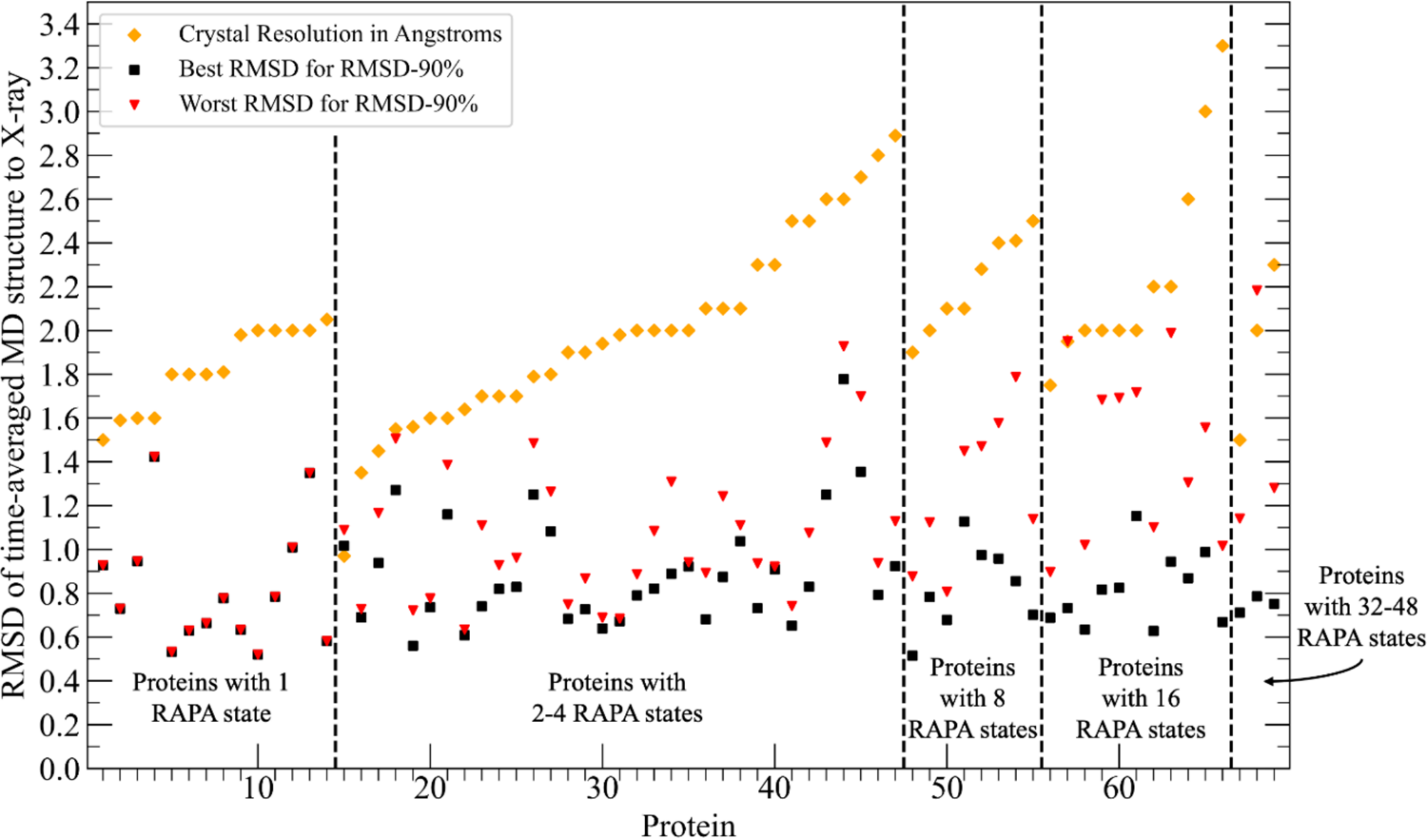
RMSD between time average MD structure and X-ray coordinates. Best RMSD (black squares) and worst RMSD (red triangles) are shown for each of the 69 targets. X-ray crystal structure resolution is shown for each target (yellow diamonds). Data shown is for the RMSD-90% subset of atoms (Section 2.3.2.2). Data for a subset of the systems in which RMSDs were calculated for unrestrained simulations is shown in Figure S6 in the Supporting Information.

**Figure 8:**
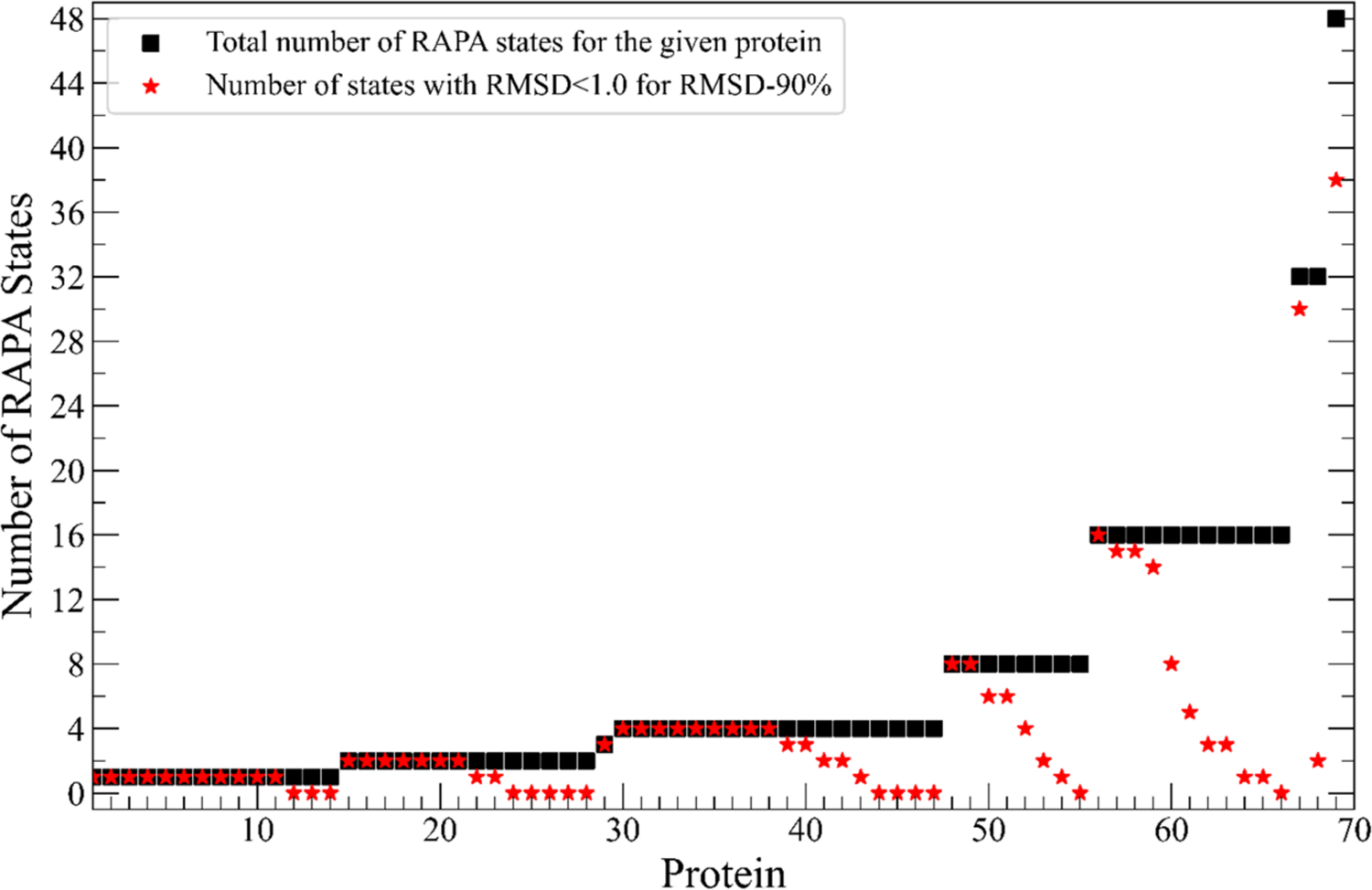
Number of RAPA proposed configurations for 69 systems. The initial number of states (black squares) and number of those with RMSD less than 1 Å for the *RMSD-90%* subset of atoms (Section 2.3.2.2) (red stars). For many systems there are multiple RAPA proposed configurations that are consistent with the X-ray structure. Comparative data for RMSD-90%, 70%, and 50% can be found in Supporting Information (Figure S3).

#### 3.2.2 Sampling RAPA Rotamer States in Molecular Dynamics

In this section we evaluate the consistency of energetically *degenerate RP* states identified by RAPA with MD simulations. To assess this, for each system that was predicted to have *degenerate* rotamer states, we ran short (10 ns) MD simulations in which the rotamer states of *degenerate* residues were unrestrained and allowed to freely interchange. For each *degenerate* residue the rotamer states were arbitrarily labeled “A” and “B” and separate MD simulations were run in which the initial rotamer states were initialized in either “A” or “B”. In these simulations, we tracked the dihedral angles for each *degenerate* residue and characterized three possible outcomes

##### 3.2.2.1 Interchange

Both rotamer states are sampled. In these simulations, the rotamer states of both “A” and “B” were sampled regardless of whether the MD simulations had initial rotamer states corresponding to “A” or “B” (Figure 9—top). In this case, the RAPA prediction was considered consistent with the MD simulations as both rotamer states were sampled in the MD simulations.

**Figure 9:**
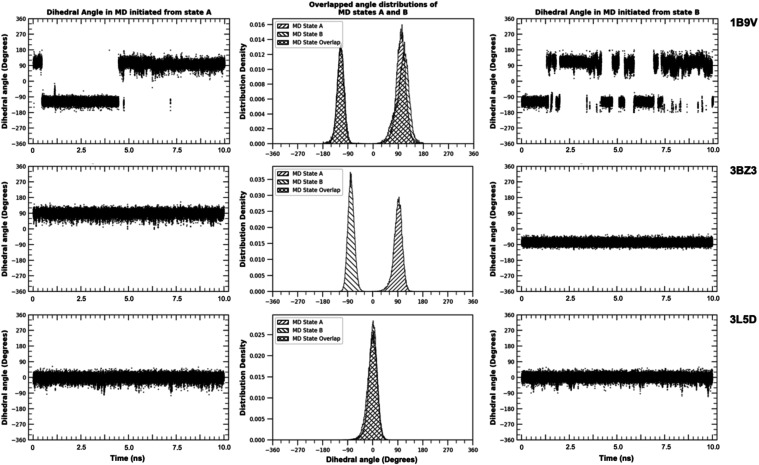
Interchange of Rotamer States: Dihedral angles vs time for MD simulations that started in state “A” (left) and state “B” (right). The middle figures have distributions obtained from the simulations that were initiated in rotamer state “A” (shaded in forward-slash lines) and state “B” (shaded in back-slash lines lines). The data is for residue ASN-230 (PDB: 1B9V^54^), GLN-624 (PDB: 3BZ3 ^55^), and ASN-294 (PDB: 3L5D^41^). RAPA-predicted degenerate rotamer states for each of these residues. This is consistent for the top two residues however for ASN-294 in 3L5D, the MD simulation revealed that only one state is stable. Data that appears cross-hatched in the middle figures because the forward and backlash lines from the distributions of each simulation overlap. Figures S8–S10 in Supporting Information shows the interactions of the residues in the different rotamer states.

##### 3.2.2.2 Only the Initial State Is Sampled

In these simulations, no transitions between rotamer states were observed in the MD simulations. Rotamer states were found to remain in the state they were initialized in (Figure 9—middle). In these cases, both rotamer states are considered *stable* over the course of the MD simulation and we assess that the RAPA prediction of *degenerate* states is correct.

##### 3.2.2.3 Only One State Is Stable

In these simulations, the rotamer states of the RAPA-predicted *degenerate* residues switched to a single state and remained there for the entirety of the MD simulation (Figure 9—bottom). In these cases, the residues switched rotamer state during minimization, equilibration, or within the first nanosecond of the production simulation. In this case, the RAPA prediction of degenerate rotamer states is considered inconsistent with the MD simulation or incorrect.

##### 3.2.2.4 *Degenerate* ASN/GLN Rotamer Consistency with Molecular Dynamics Simulations

Of the 87 residues that RAPA predicted to be degenerate, 49 residues (56%) had two stable states in the MD simulations either sampling both states (24/49) or having no interchange between the two states (25/49). In these cases, the predictions were considered consistent with the MD simulations and the RAPA predictions were assessed to be correct. On the other hand, for the other 38 residues (44%), only one state was found to be stable in the dynamic simulations and the RAPA predictions were assessed to be incorrect (Table 2).

**Table 2:** MD Analysis of *Degenerate* ASN and GLN Residues

# of degenerate residues	# of degenerate residues correctly predicted by RAPA in MD	# of residues outcome #1	# of residues outcome #2	# of residues outcome #3
51 ASN	22	13	9	29
36 GLN	27	11	16	9

##### 3.2.2.5 *Degenerate* HIS Rotamer and Protonation State MD Analysis

Seventy-nine HIS residues were predicted to be *degenerate*. Of these, 73 (92%) were found to be stable in each of the multiple degenerate states predicted by RAPA in their respective MD simulations (Table 3). We note that we considered a protonation state to be unstable if the rotamer state moved at least 30 degrees away from the predicted stable state.

**Table 3:** MD Analysis of *Degenerate* RAPA-Predicted HIS Residues

HIS residues degeneracy	# RAPA-predicted degenerate residues	# residues inherently *stable* in their *degenerate* states over the course of the MD simulations	# residues stable in only one of the RAPA-predicted states
two rotamer states	20	18	2
two protonation states	16	14	2
two states^a^	35	34	1
more than two states^a^	8	7	1^b^

a Corresponds to states that differ in both rotamer and protonation state.

b For HIS159 in 1D3G, three states were predicted to be *degenerate* however only two were stable in MD.

## 4 Discussion

The inability of X-ray scattering experiments to resolve hydrogens and differentiate between oxygen, carbon, and nitrogen atoms leaves ambiguity as to the *correct* configuration of protein side chains. To address this a number of methods have been developed that attempt to identify the precise atomic configuration, however, most of these methods identify a single configuration. When, in fact, there may not be one correct configuration but multiple configurations that are found in protein crystals. Here, we have introduced a protocol, RAPA, that attempts to identify all configurations of the protein that are energetically accessible and consistent with the resolved X-ray data. The protocol does this by examining the local h-bond networks of *ambiguous* side chains and quickly approximates the energetics of potential alternate configurations. When implemented computationally, the RAPA protocol takes several minutes to fully output a set of structures that are consistent with the X-ray structure and determined to be energetically *degenerate.*

We applied the RAPA protocol to 77 protein structures and identified that 63 of these had multiple potential configurations resulting in a total of 469 configurations. In short 10 ns MD simulations, 467 of these configurations maintained conformations close to the crystal structure with time-averaged RMSDs within 2.0 Å or less (*RMSD-90%*). These results suggest that the structures produced by RAPA are stable and consistent with their crystal structures. We also evaluated RAPA’s predictions of *degenerate* rotamer states of ASN, GLN, and HIS residues in 10 ns MD simulations in which the rotamer states were unrestrained and found that 56% of the ASN/GLN and 92% of the HIS had two or more stable states matching these predictions.

The fundamental premise of structure-based drug discovery is that a small molecule lead needs to be electrostatically complementary to the protein surface making h-bond or hydrophobic contacts where appropriate. When the rotamer or protonation states of *ambiguous* residues change, the positions or character of these contacts change and, correspondingly, the chemical matter that is complementary to the surface differs. Limiting computer aided drug discovery workflows to a single configuration of a protein effectively limits the search for viable leads to chemical matter that is complementary to that configuration. The RAPA protocol addresses this by identifying multiple configurations for most of the systems studied, each of which has the potential to bind unique chemical matter. Conversely, exploring every possible configuration of these side chains results in a combinatorial explosion and, thus, investigating all possible configurations is intractable in a computer aided drug discovery workflow. The RAPA protocol addresses this problem by efficiently identifying only those configurations that are energetically accessible and discarding those that are not. Importantly, for 62 of the 77 systems investigated here, 8 or fewer energetically accessible configurations were identified, and the largest number of such *degenerate* configurations was 24 per monomer. This suggests that investigating all configurations predicted by RAPA can be feasible in a drug discovery workflow context and expand the chemical space of compounds to those that are complementary to not only a single configuration but those that are complementary to multiple configurations.

The RAPA protocol introduced here is designed to identify energetically competitive configurations of a protein each with alternate subsets of rotamer and protonation states. Here, we have shown that we can identify multiple configurations for each protein that are stable and have structures that are consistent with the experimental crystal structures in MD simulations. This initial evaluation of the protocol used a relatively simple estimator of residue–residue energetics which can be improved upon in a variety of ways, these include, but are not limited to calculating residue specific corrections based on environmental estimates of p*K*_a_, inclusion of an entropy term to obtain a free energy estimate of the relative populations of alternate RAPA states, a more extensive treatment of rotational flexibility and possible hydrogen positions in hydroxy and thiol groups, and using explicit quantum mechanical calculations of each distinct residue–residue interaction for the energetic estimators. Each of these may lead to better success in identifying accessible alternate RAPA configurations. There is also the question of how to prioritize RAPA configurations. Depending on the expense of the application, one may wish to prioritize some RAPA configurations over others. For example running a virtual screen on 8 configurations may be feasible but not on 1024. One way to prioritize configurations is by ranking them by order by RMSD from the crystal structure with lower values being prioritized over higher values. While this may prove to be effective, it is slow in that it requires an MD simulation for each proposed RAPA configuration. Another faster approach is to track the sum of the energy differences between each alternate rotamer states and prioritize based on the total energy estimate of each complete RAPA configuration. For example, the highest priority state would be the one in which every “*degenerate”* RAPA state is in the lower energy configuration.

## Supplementary Material



## Data Availability

Open Eye Spruce-prepared configurations, the RAPA output configurations for the set of 77 systems, and input files sufficient to reproduce the molecular dynamics simulations are available on the KurtzmanLab github: https://github.com/KurtzmanLab/RAPAData. The PDBIDs for all proteins are listed in Table S1 in the Supporting Information. These are available online RCSB PDB: Homepage. Software used to generate the results (Amber Tools, AMBER, Open eye Tools, Schrodinger) is commercial. Software implementing the RAPA protocol is opensource under the MIT License and available at: https://github.com/Ventus-Therapeutics/rapa/tree/v1.0

## Footnotes

1 We use the term “configuration” to denote a complete description of the positions of every atom in the protein.

2 We use the italicized term “*ambiguous*” to denote that the protonation/rotamer state is not yet determined as *known* by the RAPA protocol.

## Notes

Additional details on computations, molecular examples of rotamer states, additional data for validation simulations, raw data for figures, and code timing (PDF)
