# Supplementary material for: A Self-Consistent Approach to Rotamer and Protonation State Assignments (RAPA): Moving Beyond Single Protein Configurations
Source: J Chem Inf Model. 2025 Jun 11;65(14):7639–50. doi: 10.1021/acs.jcim.5c00859 (PMC12308802; doi:10.1021/acs.jcim.5c00859)
Supplement: Supplementary file 1 [file ci5c00859_si_001.pdf]

A Self Consistent Approach to Rotamer and Protonation State Assignments (RAPA): Moving Beyond Single Protein Configurations

Supporting Information

Mossa Ghattas[1,2], Prerna Gera[4], Steven Ramsey[3], Anthony Cruz Balberdy[5], Nathaniel Abraham[5], Vjay Molino[2], Daniel McKay[4], Tom Kurtzman[1,2,3]

[1] Ph.D. Program in Chemistry, The Graduate Center, City University of New York, New York, USA

[2]Ph.D. Program in Biochemistry, The Graduate Center, City University of New York, New York, USA

[3] Department of Chemistry, Lehman College, City University of New York, New York, USA

[4] Ventus Therapeutics, Inc. 4800 rue Levy, Montreal, Quebec H4R 2P7, Canada

[5] Ventus Therapeutics U.S. Inc. 100 Beaver St. Suite 201, Waltham, MA 02453, USA

H-bond Energy Evaluations

We estimated the energies of hydrogen bonds using a lookup table based on heavy atom – heavy atom (hv–hv) distances and heavy atom – hydrogen – heavy atom (hv–h–hv) angles. The lookup table was constructed using a potential energy surface (PES) calculation generated using Schrodinger Jaguar[1] on a set of configurations of a water dimer with Oxygen-Oxygen (O-O) distances ranging from 2.5Å - 4.0Å with 0.1Å intervals and O···H-O angle ranging from 90°-180° with 0.1° intervals. We used B3LYP as the exchange-correlation functional theory and CC-PVTZ-PPpp as the basis set in the PES calculation. In RAPA, local hydrogen bond energies for the protein use the hv-hv distance and hv-h-hv angle as a proxy for the O-O distance and O···H-O angle (**Figure S1**).

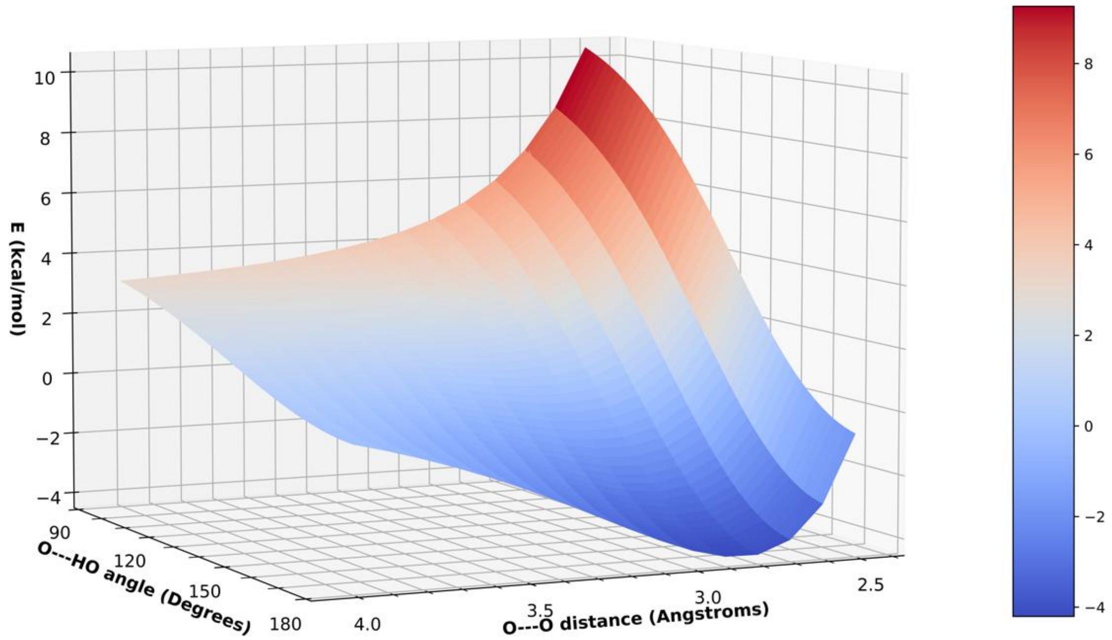

Figure S1. The Potential Energy Surface (PES) of a water dimer. Oxygen-Oxygen (O-O) distances ranging from 2.5Å - 4.0Å with 0.1Å intervals and O···H-O angle ranging from 90°-180° with 0.1° intervals.

Evaluation of Acid Dyads: A Case Example - HIV Protease

In the RAPA protocol neighboring carboxylate residues are examined for protonation, as the acid dyad requires one of the two residues to be protonated. To accomplish this each possible carboxylate oxygen is protonated and

the energy evaluated. In this example, HIV protease (PDB Entry 2P3B[2]) features an acid dyad in its binding pocket (**Figure S2**). Here, environment A and B show H-bond interactions (whereas the other protonation states – not shown – have electrostatic clashes). The energetics of A &B are within 1 kcal of energy (delta energy of 0.11 kcal) and are therefore considered *degenerate*.

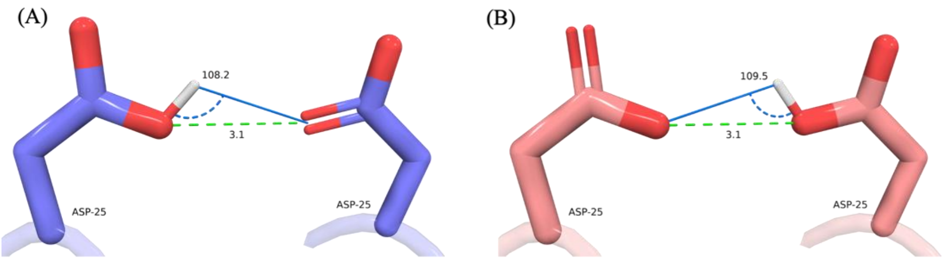

Figure S2. The interactions of ASP25-ASP25 dyad in HIV Protease (PDB entry 2P3B[2]). Panels A and B show the two viable protonation states for ASP dyad. The O-O distance (green dashed line) and the O-H---O angles (marine line and arc) are shown. The ASP dyad is formed from residues 25 from chains A & B from the pdb.

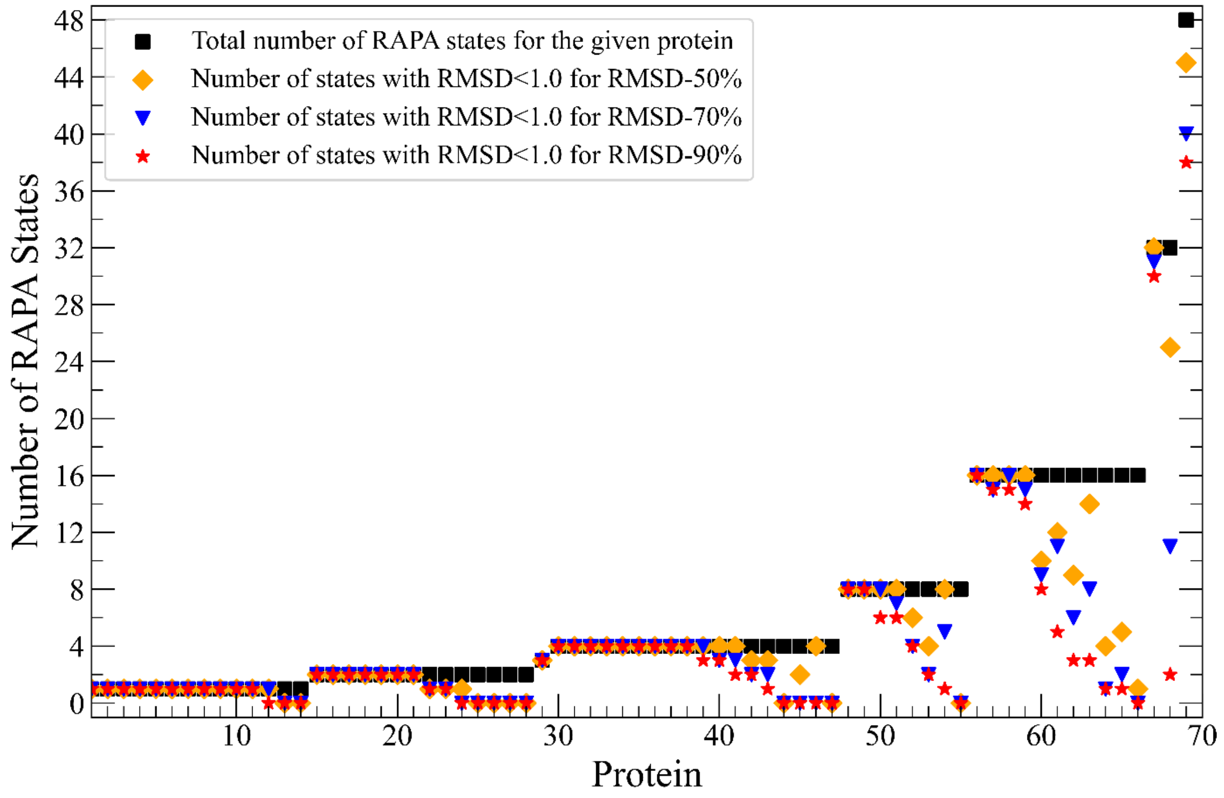

Figure S3. Number of RAPA states for 69 systems. The initial number of RAPA states (black squares), number of those with RMSD less than 1Å for the *RMSD-50%* subset of atoms (yellow diamonds), *RMSD-70%* (blue triangles), *RMSD-90%* (red stars) are shown for each target. Data is for the 3DS core restrained MD simulations.

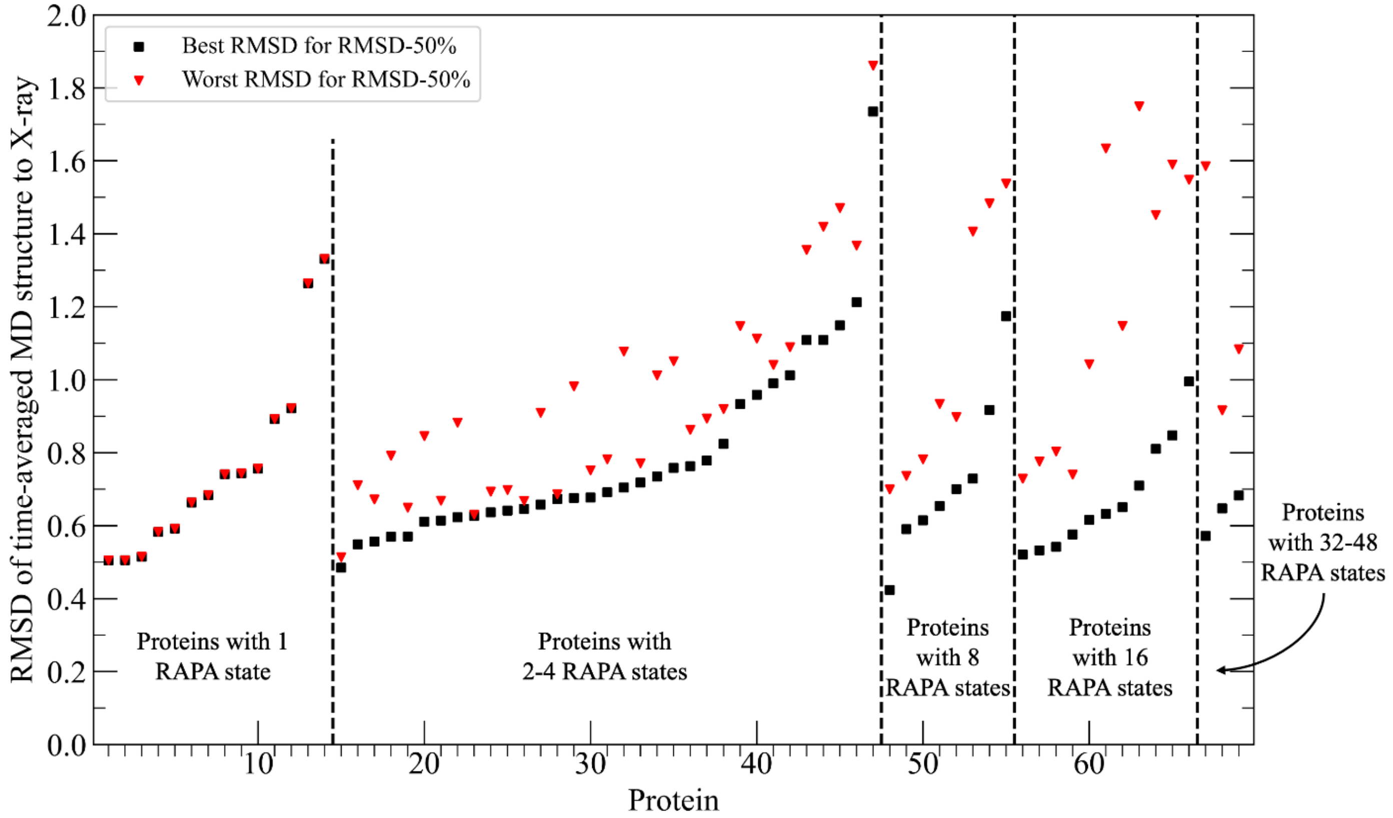

Figure S4. RMSD between time average MD structure and X-Ray coordinates: Best RMSD (black squares) and worst RMSD (red triangles) are shown for each of the 69 targets for the *RMSD-50%* subset of atoms. Data is for the 3DS core restrained MD simulations.

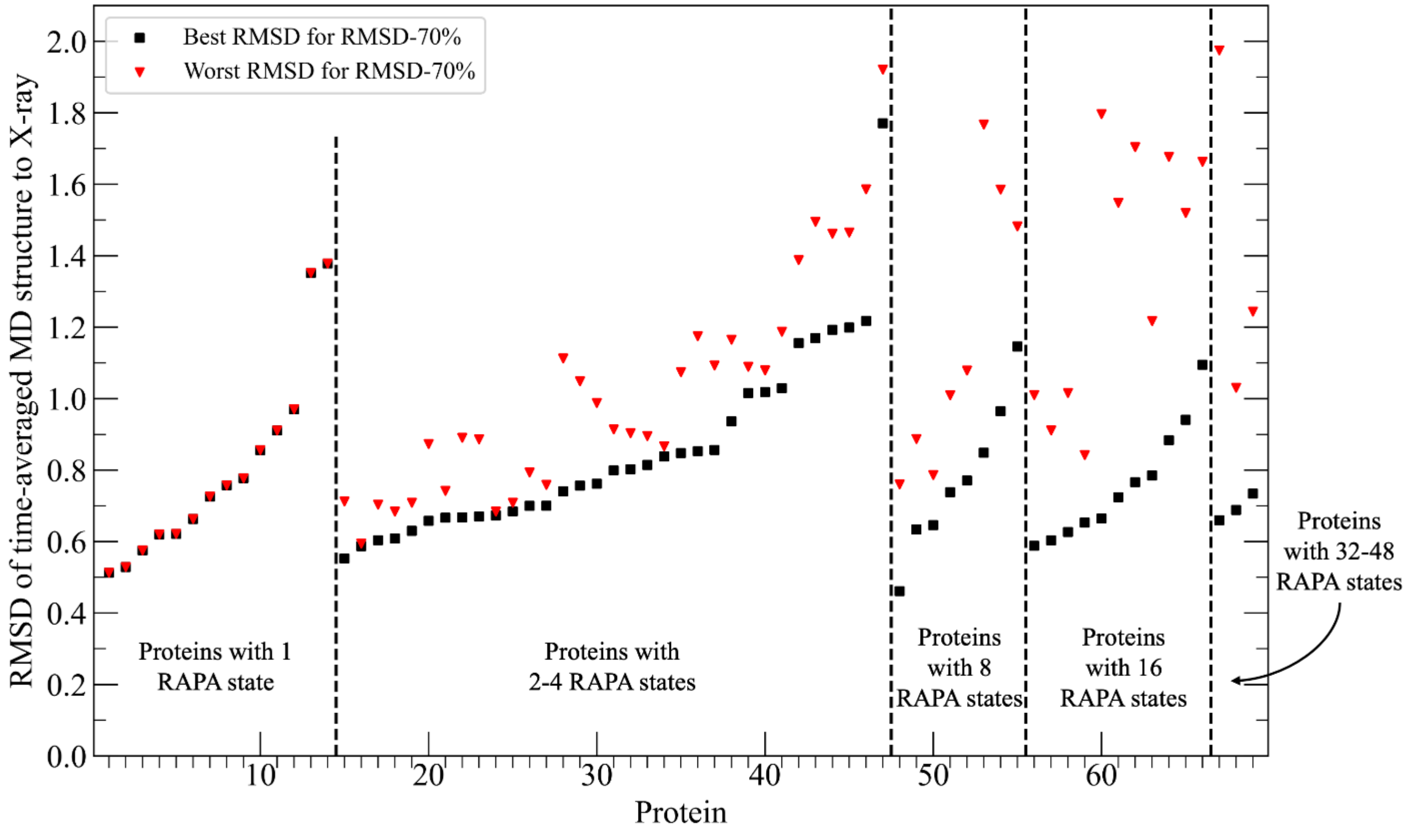

Figure S5. RMSD between time average MD structure and X-Ray coordinates: Best RMSD (black squares) and worst RMSD (red triangles) are shown for each of the 69 targets for the *RMSD-70%* subset of atoms. Data is for the 3DS core restrained MD simulations.

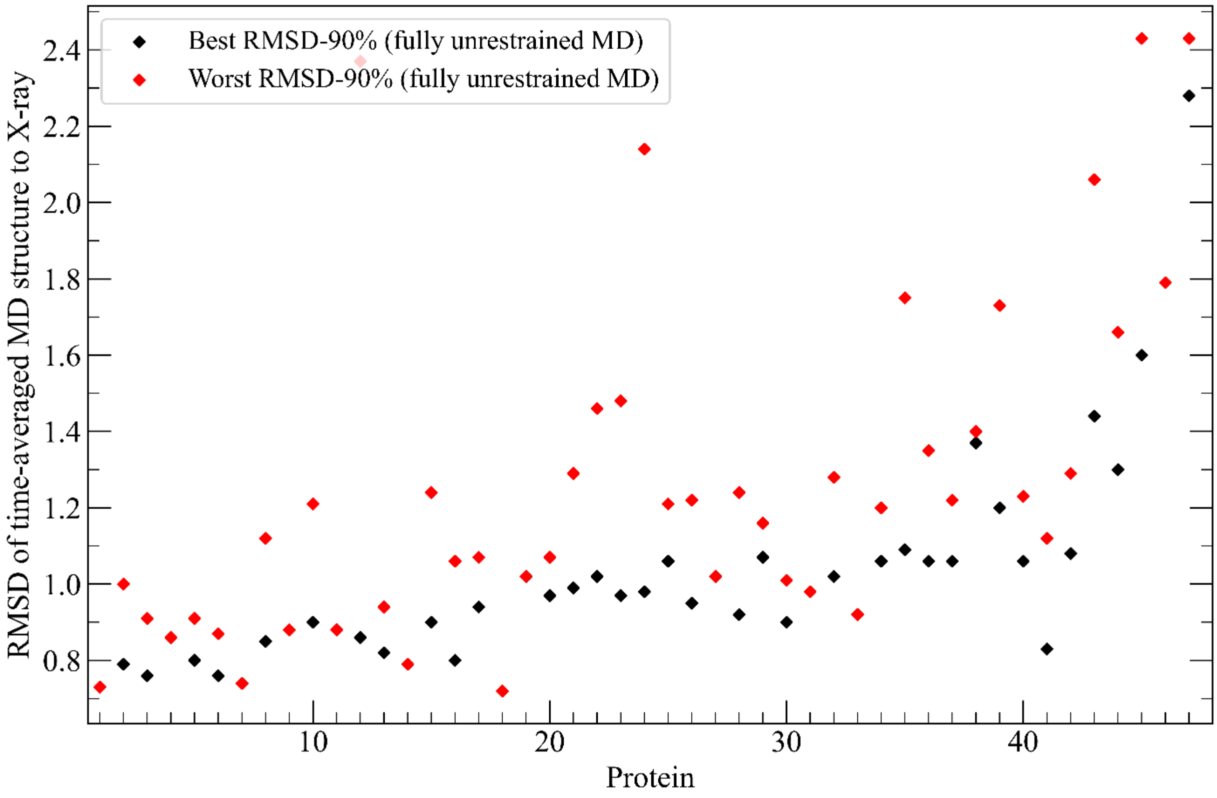

Figure S6: RMSD between time average MD structure and X-Ray coordinates: Best RMSD (black diamonds) and worst RMSD (red diamonds) are shown for each of the 69 targets for the *RMSD-70%* subset of atoms. Data is for the unrestrained MD simulations.

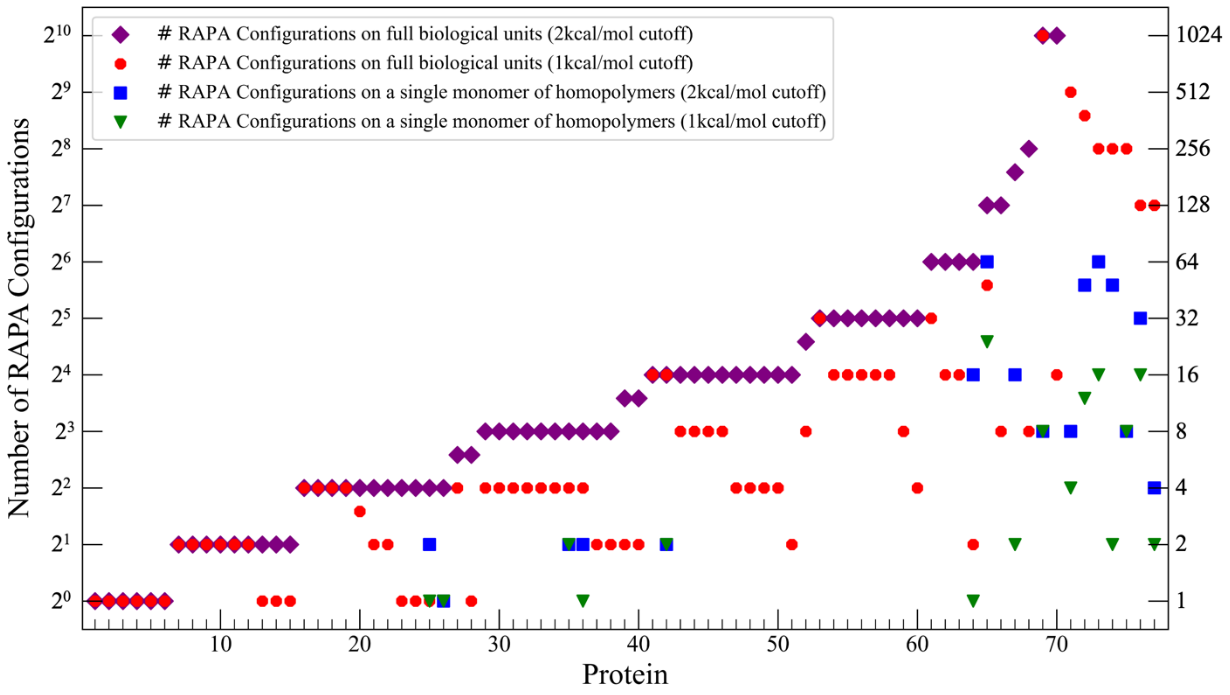

Figure S7. A comparison of the total number of unique configurations determined by RAPA for 77 protein systems with cutoff energies of 1 and 2 kcal/mol. The full biological unit of the system is evaluated at a cutoff of 1kcal/mol (red circles), 2 kcal/mol (purple diamonds). For the single repeating monomer at a cutoff of 1 kcal/mol (green diamonds) and 2kcal/mol (blue squares).

Figures S8 through S10 show the rotamer states for each of the three systems from Figure 9 in the main text Section 3.2.2 (Sampling of RAPA Rotamer States in Molecular Dynamics) of the main body of text.

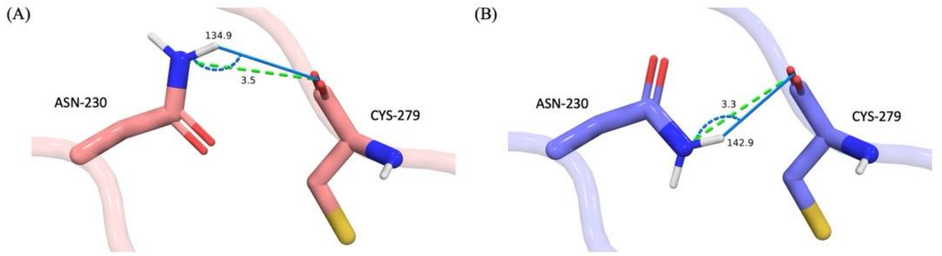

Figure S8. The interactions of ASN-230 residue in influenza virus neuraminidase structure (PDB entry 1B9V[3]). RAPA evaluates both rotamer states to be considered energetically viable for this residue. Hydrogen bond interactions (green dashed line), and their angles (marine line and arc) are shown.

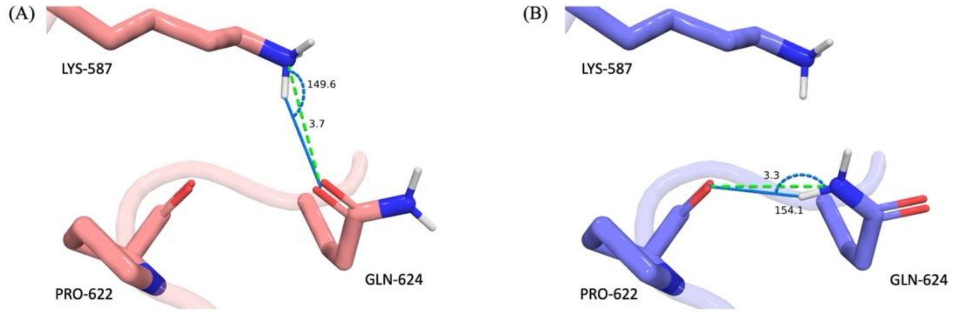

Figure S9. The interactions of GLN-624 residue in focal adhesion kinase structure (PDB entry 3BZ3[4]). RAPA evaluates both rotamer states to be considered energetically viable for the 1kcal/mol energetic cutoff for this residue. Hydrogen bond interactions (green dashed line), and their angles (marine line and arc) are shown.

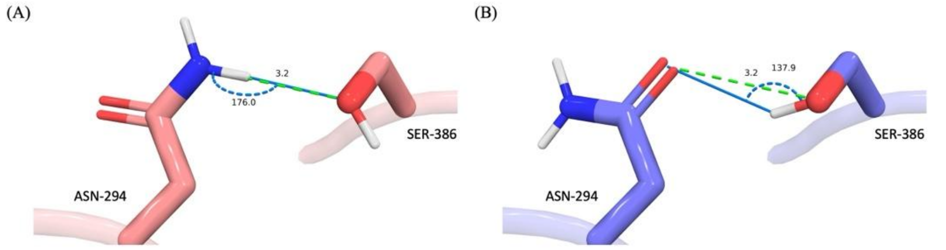

Figure S10. The interactions of ASN-294 residue in beta-site amyloid cleaving enzyme structure (PDB entry 3L5D[5]). RAPA evaluates both rotamer states to be considered energetically *degenerate* for this residue as they are within 1kcal of each other. Hydrogen bond interactions (green dashed line), and their angles (marine line and arc) are shown. Note that while RAPA predicted that these two states were *degenerate*, the prediction is inconsistent with the MD simulation in which only one state (State A) is well sampled. The simulation starts from state B, it flipped into state A and did not return to state B.

Table S1: Raw Data for **Figure 6.** The total number of unique configurations determined by RAPA for 77 protein systems with 1 kcal energy cutoff.

Protein No.	Full biological_1kcalmol	PDBID	Single monomer_1kcalmol
1	1	1NJS	
2	1	2NNQ	
3	1	2OJ9	
4	1	3G0E	
5	1	3HL5	
6	1	3MAX	
7	1	1J4H	
8	1	2AZR	
9	1	2HV5	
10	1	1H00	
11	1	1YPE	
12	1	1UYG	
13	1	3D0E	1
14	1	1XL2	1
15	2	2E1W	
16	2	2VT4	
17	2	3BWM	
18	2	3HMM	
19	2	3NXO	
20	2	830C	
21	2	3EML	
22	2	3KL6	
23	2	2AM9	
24	2	2OF2	
25	2	3EL8	
26	2	3KRJ	
27	2	2HZI	
28	2	2ETR	1
29	3	1D3G	
30	4	1L2S	
31	4	1UDT	
32	4	2AYW	
33	4	3PBL	
34	4	2ICA	
35	4	3E37	
36	4	1SQT	
37	4	2GTK	
38	4	2P54	
39	4	1LRU	
40	4	3EQH	
41	4	2QD9	
42	4	3LPB	
43	4	2ZDT	
44	4	3L3M	
45	4	3D4Q	
46	4	1MV9	2
47	4	3KGC	1
48	8	2OWB	
49	8	3BQD	
50	8	3M2W	
51	8	3CQW	
52	8	1BCD	
53	8	2P2I	
54	8	3CJO	
55	8	3CHP	
56	16	3KBA	
57	16	2AA2	
58	16	3BZ3	
59	16	3G6Z	
60	16	2I0E	
61	16	3F07	
62	16	2OJG	
63	16	3L5D	
64	16	2ZNP	
65	16	1KVO	2
66	16	2FSZ	2
67	32	2OI0	
68	32	3F9M	
69	48	3FRJ	24
70	128	3NF7	16
71	128	4TRJ	2
72	256	1E66	16
73	256	2B8T	8
74	256	1B9V	2
75	384	1SYN	12
76	512	3CCW	4
77	1024	3BGS	8

Table S2: Raw Data for **Figure 7.** Data shown is for the RMSD-90% subset of atoms for the 3DS core restrained MD simulations.

Protein No.	Number of RAPA states	PDBID	Crystal Resolution	Best_rmsd_RMSD-90	Worst_rmsd_RMSD-90
1	1	1XL2	1.50	0.93	0.93
2	1	2HV5	1.59	0.73	0.73
3	1	1H00	1.60	0.95	0.95
4	1	3G0E	1.60	1.42	1.42
5	1	1J4H	1.80	0.53	0.53
6	1	3HL5	1.80	0.63	0.63
7	1	2NNQ	1.80	0.66	0.66
8	1	1YPE	1.81	0.78	0.78
9	1	1NJS	1.98	0.63	0.63
10	1	2AZR	2.00	0.52	0.52
11	1	1UYG	2.00	0.78	0.78
12	1	2OJ9	2.00	1.01	1.01
13	1	3D0E	2.00	1.35	1.35
14	1	3MAX	2.05	0.58	0.58
15	4	2AYW	0.97	1.02	1.09
16	2	3NXO	1.35	0.69	0.73
17	2	3KL6	1.45	0.94	1.17
18	4	3KGC	1.55	1.27	1.51
19	4	2ICA	1.56	0.56	0.72
20	3	1D3G	1.60	0.74	0.78
21	2	830C	1.60	1.16	1.39
22	2	2AM9	1.64	0.61	0.63
23	2	3HMM	1.70	0.74	1.11
24	4	2QD9	1.70	0.82	0.93
25	2	2HZI	1.70	0.83	0.96
26	4	2P54	1.79	1.25	1.49
27	4	3E37	1.80	1.08	1.26
28	4	1SQT	1.90	0.68	0.75
29	4	1MV9	1.90	0.73	0.87
30	4	1L2S	1.94	0.64	0.69
31	2	3BWM	1.98	0.67	0.68
32	4	3EQH	2.00	0.79	0.89
33	4	3LPB	2.00	0.82	1.08
34	4	2ZDT	2.00	0.89	1.31
35	2	2OF2	2.00	0.92	0.94
36	4	2GTK	2.10	0.68	0.89
37	4	1LRU	2.10	0.87	1.24
38	2	3KRJ	2.10	1.04	1.11
39	4	1UDT	2.30	0.73	0.94
40	2	3EL8	2.30	0.91	0.92
41	2	2E1W	2.50	0.65	0.74
42	4	3L3M	2.50	0.83	1.08
43	2	3EML	2.60	1.25	1.49
44	2	2ETR	2.60	1.78	1.93
45	2	2VT4	2.70	1.35	1.70
46	4	3D4Q	2.80	0.79	0.94
47	4	3PBL	2.89	0.92	1.13
48	8	1BCD	1.90	0.51	0.88
49	8	3CQW	2.00	0.78	1.12
50	8	3CHP	2.10	0.68	0.81
51	8	2OWB	2.10	1.13	1.45
52	8	3CJO	2.28	0.97	1.47
53	8	2P2I	2.40	0.96	1.58
54	8	3M2W	2.41	0.86	1.79
55	8	3BQD	2.50	0.70	1.14
56	16	3L5D	1.75	0.69	0.90
57	16	2AA2	1.95	0.73	1.95
58	16	3KBA	2.00	0.63	1.02
59	16	3G6Z	2.00	0.82	1.68
60	16	2OJG	2.00	0.82	1.69
61	16	1KVO	2.00	1.15	1.72
62	16	3BZ3	2.20	0.63	1.10
63	16	2FSZ	2.20	0.94	1.99
64	16	2I0E	2.60	0.87	1.31
65	16	2ZNP	3.00	0.99	1.56
66	16	3F07	3.30	0.67	1.02
67	32	3F9M	1.50	0.71	1.14
68	32	2OI0	2.00	0.79	2.18
69	48	3FRJ	2.30	0.75	1.28

Table S3: Raw Data for **Figure 8.** Data shown is for the RMSD-90% subset of atoms for the 3DS core restrained MD simulations.

Protein No.	PDBID	Number of RAPA states	Number states below 1A RMSD-90
1	1XL2	1	1
2	2HV5	1	1
3	1H00	1	1
4	1J4H	1	1
5	2NNQ	1	1
6	3HL5	1	1
7	1YPE	1	1
8	1NJS	1	1
9	1UYG	1	1
10	2AZR	1	1
11	3MAX	1	1
12	3G0E	1	0
13	2OJ9	1	0
14	3D0E	1	0
15	3NXO	2	2
16	2AM9	2	2
17	2HZI	2	2
18	3BWM	2	2
19	2OF2	2	2
20	3EL8	2	2
21	2E1W	2	2
22	3KL6	2	1
23	3HMM	2	1
24	830C	2	0
25	3KRJ	2	0
26	2ETR	2	0
27	3EML	2	0
28	2VT4	2	0
29	1D3G	3	3
30	2ICA	4	4
31	2QD9	4	4
32	1MV9	4	4
33	1SQT	4	4
34	1L2S	4	4
35	3EQH	4	4
36	2GTK	4	4
37	1UDT	4	4
38	3D4Q	4	4
39	1LRU	4	3
40	3L3M	4	3
41	2ZDT	4	2
42	3LPB	4	2
43	3PBL	4	1
44	2AYW	4	0
45	3KGC	4	0
46	2P54	4	0
47	3E37	4	0
48	1BCD	8	8
49	3CHP	8	8
50	3CQW	8	6
51	3BQD	8	6
52	3M2W	8	4
53	2P2I	8	2
54	3CJO	8	1
55	2OWB	8	0
56	3L5D	16	16
57	3KBA	16	15
58	3F07	16	15
59	3BZ3	16	14
60	3G6Z	16	8
61	2OJG	16	5
62	2AA2	16	3
63	2I0E	16	3
64	2FSZ	16	1
65	2ZNP	16	1
66	1KVO	16	0
67	3F9M	32	30
68	2OI0	32	2
69	3FRJ	48	38

Table S4. Raw Data for Figure S6. RMSD between time average MD structure and X-Ray coordinates for unrestrained simulations: Best RMSD and worst RMSD are shown for each of 47 targets for the *RMSD-90%* subset of atoms. These 47 are a subset of the 69 investigated for the 3DS core restrained simulations. Data is for the unrestrained MD simulations.

Protein No.	PDBID	Number of RAPA states	Best_rmsd_RMSD-50	Worst_rmsd_RMSD-50
1	1J4H	1	0.51	0.51
2	2AZR	1	0.51	0.51
3	3MAX	1	0.51	0.51
4	3HL5	1	0.58	0.58
5	1NJS	1	0.59	0.59
6	2NNQ	1	0.66	0.66
7	2HV5	1	0.68	0.68
8	1YPE	1	0.74	0.74
9	1UYG	1	0.74	0.74
10	1H00	1	0.76	0.76
11	1XL2	1	0.89	0.89
12	2OJ9	1	0.92	0.92
13	3G0E	1	1.26	1.26
14	3D0E	1	1.33	1.33
15	2AM9	2	0.48	0.51
16	2ICA	4	0.55	0.71
17	1SQT	4	0.56	0.67
18	1UDT	4	0.57	0.79
19	1L2S	4	0.57	0.65
20	3D4Q	4	0.61	0.85
21	2OF2	2	0.61	0.67
22	2GTK	4	0.62	0.88
23	3EQH	4	0.63	0.63
24	2E1W	2	0.64	0.69
25	1D3G	3	0.64	0.70
26	3NXO	2	0.65	0.67
27	3L3M	4	0.66	0.91
28	3BWM	2	0.67	0.69
29	3LPB	4	0.68	0.98
30	1MV9	4	0.68	0.75
31	2P54	4	0.69	0.78
32	3HMM	2	0.70	1.08
33	3EL8	2	0.72	0.77
34	3PBL	4	0.73	1.01
35	2ZDT	4	0.76	1.05
36	2QD9	4	0.76	0.86
37	2HZI	2	0.78	0.89
38	1LRU	4	0.82	0.92
39	3KL6	2	0.93	1.15
40	3E37	4	0.96	1.11
41	3KRJ	2	0.99	1.04
42	2AYW	4	1.01	1.09
43	830C	2	1.11	1.36
44	2VT4	2	1.11	1.42
45	3KGC	4	1.15	1.47
46	3EML	2	1.21	1.37
47	2ETR	2	1.74	1.86
48	1BCD	8	0.42	0.70
49	3BQD	8	0.59	0.74
50	3CHP	8	0.61	0.78
51	3CJO	8	0.65	0.93
52	3CQW	8	0.70	0.90
53	3M2W	8	0.73	1.41
54	2P2I	8	0.92	1.48
55	2OWB	8	1.17	1.54
56	3KBA	16	0.52	0.73
57	3F07	16	0.53	0.78
58	3BZ3	16	0.54	0.80
59	3L5D	16	0.58	0.74
60	2I0E	16	0.62	1.04
61	2AA2	16	0.63	1.63
62	2OJG	16	0.65	1.15
63	3G6Z	16	0.71	1.75
64	2ZNP	16	0.81	1.45
65	2FSZ	16	0.85	1.59
66	1KVO	16	0.99	1.55
67	2OI0	32	0.57	1.59
68	3F9M	32	0.65	0.92
69	3FRJ	48	0.68	1.08

Table S5 (below). Subset of 77 crystal protein structures investigated by RAPA. X-ray diffraction resolution, spruce-prepared chains of the structures used by RAPA, number of RAPA proposed structures, and RAPA time performance are highlighted. The RAPA code was run on a desktop computer utilizing Intel® Core™ i7-5820K CPU @ 3.30 GHz with 64 GB of RAM. The RAPA code used one core for the analysis and wall-clock time is reported in minutes.

PDB Entry	Chain (Spruced prepared)	X-ray Diffraction Resolution (Å)	# of RAPA Proposed Configurations	RAPA Job Time (Minutes)
1H00	AaltA	1.60	1	1.1
1J4H	A	1.80	1	0.4
1NJS	A	1.98	1	2.2
1UYG	A	2.00	1	0.9
1XL2	AB	1.50	1	0.6
1YPE	HIL	1.81	1	1.3
2AZR	A	2.00	1	1.8
2HV5	A	1.59	1	1.4
2NNQ	A	1.80	1	0.5
2OJ9	A	2.00	1	0.8
3D0E	AB	2.00	1	2.6
3G0E	A	1.60	1	1
3HL5	A	1.80	1	1.2
3MAX	BaltB	2.05	1	8.6
2AM9	AaltB	1.64	2	0.9
2E1W	A	2.50	2	4.8
2ETR	AB	2.60	2	3.4
2HZI	B	1.70	2	1.3
2OF2	A	2.00	2	1
2VT4	A	2.70	2	0.7
3BWM	A	1.98	2	0.7
3EL8	A	2.30	2	0.9
3EML	A	2.60	2	1.7
3HMM	A	1.70	2	1.9
3KL6	AB	1.45	2	2.1
3KRJ	A	2.10	2	1.4
3NXO	AaltB	1.35	2	0.7
830C	B	1.60	2	7.4
1D3G	AaltA	1.60	3	2.5
1L2S	BaltB	1.94	4	1.7
1LRU	B	2.10	4	1
1MV9	ABCD	1.90	4	2.3
1SQT	A	1.90	4	2
1UDT	A	2.30	4	4.6
2AYW	A	0.97	4	2.1
2GTK	AB	2.10	4	2.5
2ICA	A	1.56	4	0.8
2P54	AB	1.79	4	1.3
2QD9	A	1.70	4	2.7

PDB Entry	Chain (Spruced prepared)	X-ray Diffraction Resolution (Å)	# of RAPA Proposed Configurations	RAPA Job Time (Minutes)
2ZDT	A	2.00	4	2.4
3D4Q	A	2.80	4	1.3
3E37	B	1.80	4	3.4
3EQH	A	2.00	4	1.7
3KGC	AB	1.55	4	1.3
3L3M	A	2.50	4	2.3
3LPB	BaltB	2.00	4	1.4
3PBL	B	2.89	4	1.4
1BCD	A	1.90	8	6.8
2OWB	A	2.10	8	1.5
2P2I	A	2.40	8	1.9
3BQD	AB	2.50	8	2.4
3CHP	A	2.10	8	4.7
3CJO	A	2.28	8	2.8
3CQW	ACaltA	2.00	8	1.9
3M2W	AaltB	2.41	8	1.5
1KVO	AB	2.00	16	3.4
2AA2	A	1.95	16	2.6
2FSZ	AB	2.20	16	4.3
2I0E	A	2.60	16	1.6
2OJG	A	2.00	16	4.2
2ZNP	AB	3.00	16	4.4
3BZ3	A	2.20	16	2.6
3F07	A	3.30	16	9.2
3G6Z	A	2.00	16	3.3
3KBA	B	2.00	16	3.2
3L5D	B	1.75	16	3.2
2OI0	A	2.00	32	10.3
3F9M	A	1.50	32	9.1
3FRJ	AB	2.30	48	15.7
3NF7	ABaltB	1.80	128	14.8
4TRJ	ABCDsym1	1.73	128	39.4
1B9V	ABCDsym2	2.35	256	874.4
1E66	AB	2.10	256	154.9
2B8T	ABCD	2.00	256	51.4
1SYN	AB	2.00	384	75.4
3CCW	ABCD	2.10	512	371.6
3BGS	ABC	2.10	1024	305.2

Table S6 (below). RAPA analysis of ASN, GLN, and HIS residues in the 77 crystal structures. The PDBID, number of ASN, GLN, and HIS residues, as well as the RAPA labels of the residues are shown. Degenerate HIS residues are further split into 4 categories: (1) in two rotamer states, (2) in two protonation states, (3) in two states differing in both rotamer and protonation states, (4) in more than 2.

PDB Entry	Total # of ASN/GLN	# of fully solvated ASN/GLN	# of fixed Asn/Gln	# of degenerate ASN/GLN	Total # of HIS	# of fully solvated HIS	# of fixed HIS	# of degenerate HIS (Category 1)	# of degenerate HIS (Category 2)	# of degenerate HIS (Category 3)	# of degenerate HIS (Category 4)
1H00	17	2	15		10		10				
1J4H	6		6		3		3				
1NJS	17	1	16		5		5				
1UYG	16	4	12		4		4				
1XL2	18	5	13		2		2				
1YPE	19	1	18		5		5				
2AZR	24	5	19		9		9				
2HV5	28	5	23		9		9				
2NNQ	6	1	5		1		1				
2OJ9	20	1	19		3		3				
3D0E	34	4	30		18		18				
3G0E	21	3	18		6		6				
3HL5	8	2	6		5		5				
3MAX	30	6	24		13		13				
2AM9	26	3	22	1	8		8				
2E1W	22		22		11		10	1			
2ETR	52	2	49	1	12		12				
2HZI	17	3	14		6		6				
2OF2	20	4	16		4		4				
2VT4	15	5	9	1	2		2				

PDB Entry	Total # of ASN/GLN	# of fully solvated ASN/GLN	# of fixed Asn/Gln	# of degenerate ASN/GLN	Total # of HIS	# of fully solvated HIS	# of fixed HIS	# of degenerate HIS (Category 1)	# of degenerate HIS (Category 2)	# of degenerate HIS (Category 3)	# of degenerate HIS (Category 4)
3BWM	15	3	12		6		5			1	
3EL8	19	1	18		3		2			1	
3EML	39	3	36		8		7			1	
3HMM	21	5	16		10		9			1	
3KL6	22		22		6		5		1		
3KRJ	33	3	28	2	9		9				
3NXO	17	1	16		3		2			1	
830C	8		8		9		8			1	
1D3G	25	2	23		8		7				1
1L2S	41	4	35	2	5		5				
1LRU	12	2	10		4		2	1		1	
1MV9	27	4	20	3	20		20				
1SQT	18	3	13	2	9		8	1			
1UDT	39	7	32		12		10	1		1	
2AYW	26	3	22	1	3		2		1		
2GTK	25	6	19		7		5			2	
2ICA	12		12		4		3			1	
2P54	25	2	22	1	10		9			1	
2QD9	27	2	25		12		11	1			
2ZDT	33	3	30		10		9			1	

PDB Entry	Total # of ASN/GLN	# of fully solvated ASN/GLN	# of fixed Asn/Gln	# of degenerate ASN/GLN	Total # of HIS	# of fully solvated HIS	# of fixed HIS	# of degenerate HIS (Category 1)	# of degenerate HIS (Category 2)	# of degenerate HIS (Category 3)	# of degenerate HIS (Category 4)
3D4Q	26	2	23	1	9		9				
3E37	34	5	28	1	16		16				
3EQH	22	3	18	1	8		7			1	
3KGC	22	2	19	1	4		4				
3L3M	30	5	23	2	9		9				
3LPB	29	5	22	2	8	1	7				
3PBL	32	3	27	2	6		6				
1BCD	21	3	17	1	12		10		2		
2OWB	15	1	13	1	10		8			2	
2P2I	16	2	14		15		13		2		
3BQD	29	4	24	1	5		3	1		1	
3CHP	47	4	43		16		15			1	
3CJO	25	2	22	1	7		5		2		
3CQW	18	2	16		10		8		1		1
3M2W	24	4	18	2	8		8				
1KVO	18		16	2	8		7				1
2AA2	25	3	18	4	6		6				
2FSZ	28	1	25	2	18	4	12	1	1		
2I0E	21	3	17	1	8		7				1
2OJG	30	1	26	3	13		12			1	

PDB Entry	Total # of ASN/GLN	# of fully solvated ASN/GLN	# of fixed Asn/Gln	# of degenerate ASN/GLN	Total # of HIS	# of fully solvated HIS	# of fixed HIS	# of degenerate HIS (Category 1)	# of degenerate HIS (Category 2)	# of degenerate HIS (Category 3)	# of degenerate HIS (Category 4)
2ZNP	45	4	40	1	18		15	2		1	
3BZ3	21		19	2	5		5				
3F07	24	2	19	3	13		13				
3G6Z	24	2	21	1	7		7				
3KBA	27	6	17	4	6		6				
3L5D	31	8	20	3	7		7				
2OI0	25	2	22	1	8		5			3	
3F9M	28	4	24		10		6	2		1	1
3FRJ	33	10	21	2	18		15	1		1	1
3NF7	28	7	19	2	12		9			3	
4TRJ	72	12	56	4	20		20				
1B9V	72	8	60	4	48		44	4			
1E66	102	4	96	2	26		20	2		4	
2B8T	63	4	58	1	24		19		2	1	2
1SYN	54	2	48	4	22		19		1	2	
3CCW	141	13	123	5	22	2	18	2			
3BGS	72	15	51	6	24		21		3		

References

1. Bochevarov, A. D. *et al.* Jaguar: A high-performance quantum chemistry software program with strengths in life and materials sciences. *Int. J. Quantum Chem.* **113**, 2110–2142 (2013).

2. Sanches, M. *et al.* Structural Characterization of B and non-B Subtypes of HIV-Protease: Insights into the Natural Susceptibility to Drug Resistance Development. *J. Mol. Biol.* **369**, 1029–1040 (2007).

3. Finley, J. B. *et al.* Novel aromatic inhibitors of influenza virus neuraminidase make selective interactions with conserved residues and water molecules in the active site 1 1Edited by I. A. Wilson. *J. Mol. Biol.* **293**, 1107–1119 (1999).

4. Roberts, W. G. *et al.* Antitumor Activity and Pharmacology of a Selective Focal Adhesion Kinase Inhibitor, PF-562,271. *Cancer Res.* **68**, 1935–1944 (2008).

5. Zhu, Z. *et al.* Discovery of Cyclic Acylguanidines as Highly Potent and Selective β-Site Amyloid Cleaving Enzyme (BACE) Inhibitors: Part I—Inhibitor Design and Validation. *J. Med. Chem.* **53**, 951–965 (2010).
